# A Multiagent Large Language Model Framework for Emergency Treatment Recommendation in Acute Ischemic Stroke: Development and Validation Study

**DOI:** 10.2196/96304

**Published:** 2026-07-30

**Authors:** Bicong Yan, Ruipeng Zhang, Li Chen, Xinyu Song, Zhongzheng Cao, Yuehua Li

**Affiliations:** 1 Department of Diagnostic and Interventional Radiology, Shanghai Sixth People's Hospital, No.600 Yishan Road, Xuhui District, Shanghai, 200233, China, 86 18918727305; 2 Faculty of Medical Imaging Technology, College of Health Science and Technology, Shanghai Jiao Tong University, Shanghai, China

**Keywords:** acute ischemic stroke, large language model, multiagent system, decision support, clinical safety

## Abstract

**Background:**

Acute ischemic stroke (AIS) treatment selection requires rapid, guideline-concordant integration of clinical, imaging, and laboratory data, including therapeutic windows, contraindications, stroke severity, and imaging eligibility. This process is complex, expertise-dependent, and vulnerable to safety-critical errors.

**Objective:**

This study aimed to develop and validate a structured multiagent large language model (LLM) framework for AIS decision support using real-world cases and to assess its accuracy, safety, auditability, and impact on physician decision-making, particularly among junior physicians and nonspecialists.

**Methods:**

We developed a multiagent LLM framework that used structured outputs and guideline-based reasoning to generate treatment recommendations (intravenous thrombolysis, endovascular thrombectomy, standard medical therapy, or non-AIS, or nonstroke) and Trial of ORG 10172 in Acute Stroke Treatment (TOAST) classification. The framework was evaluated using multicenter retrospective real-world cases from 2 hospitals collected between January 2018 and March 2025, prospective cases from February to May 2025, and literature-derived challenging cases from PubMed between January 2024 and January 2025. Performance was assessed against clinical reference standards. Safety was assessed using omission and hallucination event rates, instruction adherence, and 5-point clinical safety ratings. In a prospective physician study, physicians with different seniority and specialty backgrounds made AIS treatment and TOAST classification decisions with and without LLM support. Physician-case decision-level outcomes were analyzed using a binomial generalized linear mixed-effects model accounting for physician and case effects.

**Results:**

The final analysis included 1055 group A cases, 721 group B cases, 144 literature-derived group C cases, and 161 prospectively collected group D cases. Across representative Baichuan, Qwen, DeepSeek, and GPT models, the multiagent framework consistently improved treatment recommendation accuracy. Model-level accuracy ranges increased from 0.546‐0.737 to 0.687‐0.851 in group A, from 0.587‐0.698 to 0.671‐0.813 in group B, and from 0.507‐0.646 to 0.667‐0.750 in group C. TOAST classification improved overall, with cohort-level variation. Across evaluated models, the multiagent framework increased the mean clinical safety score from 3.70 to 4.01 and reduced mean hallucination and omission rates from 33.6% to 20.6% and from 38.5% to 24.5%, respectively. In the prospective physician study, LLM support increased treatment decision accuracy from 73.1% to 88.6% (odds ratio 2.86, 95% CI, 2.27‐3.60; *P*<.001). Accuracy gains were largest among junior and nonspecialist physicians, including junior specialists (0.667 to 0.833), junior nonspecialists (0.600 to 0.846), and senior nonspecialists (0.667 to 0.850). TOAST classification performance also improved (odds ratio 3.63, 95% CI 2.85‐4.64; *P*<.001).

**Conclusions:**

A structured multiagent framework improved LLM performance with average improvements of 18.9% in AIS treatment recommendation and TOAST classification, while producing more structured, auditable outputs with higher safety ratings. It was associated with higher physician decision accuracy, with larger gains among less-experienced physicians, suggesting the potential to narrow expertise-related decision accuracy gaps. Prospective multicenter studies are needed to assess effects on workflow and clinical outcomes.

## Introduction

Stroke remains a leading cause of disability and death worldwide, with the greatest burden borne by low- and middle-income countries [1,2]. While acute ischemic stroke (AIS) management requires rapid and precise decision-making within narrow therapeutic windows, stroke care resources and diagnostic expertise remain profoundly uneven globally. Driven by socioeconomic inequality, geographic barriers, and workforce shortages across both resource-limited and high-income settings, these disparities lead to critical delays and misdiagnoses, worsening patient outcomes and reinforcing inequities in access to timely, specialist-level care [3-7]. These systemic challenges make equitable access to evidence-based AIS management a pressing global priority. As underscored by the World Stroke Organization Global Declaration on Stroke, which calls for equitable, evidence-based stroke systems worldwide, efforts to expand manpower and training have so far yielded only modest and uneven progress [8,9].

Large language models (LLMs) have shown potential to support clinical information synthesis and decision-making, offering a possible strategy to improve the consistency and accessibility of AIS decision support [10]. LLMs have demonstrated strong capabilities in diagnostic reasoning, differential generation, and information synthesis [11-15]. However, whether LLMs can provide reliable, guideline-concordant, and clinically safe decision support for AIS treatment selection remains insufficiently established. Most existing studies are benchmark- or theory-driven, focusing on tasks such as MedQA (United States Medical Licensing Examination) that do not adequately reflect real-world, guideline-based clinical decision-making [16,17]. This gap underscores the need for systematic case-based evaluation using real-world AIS data and physician decision-making experiments.

Our framework augments LLMs with structured clinical reasoning to support guideline-concordant therapy selection and more reliable AIS decision-making. This study had 2 objectives: to determine whether a multiagent design improves LLM performance on real-world AIS decision tasks in a multicenter, multisource evaluation, and to assess its effect on physician decision accuracy in a human-AI interaction experiment involving physicians with varying seniority and specialty backgrounds. We further examined whether LLM assistance preferentially benefits less-experienced clinicians and narrows expertise-related decision gaps, thereby supporting more consistent, safe, and accessible stroke care decision support across diverse care settings.

## Methods

### Ethical Considerations

Approval was obtained from the institutional review board of the Medical Faculty Ethics Committee of Shanghai Sixth People's Hospital affiliated to Shanghai Jiao Tong University School of Medicine (approval 2024-KY-203), and the study was registered in the Chinese Clinical Trial Registry (ChiCTR2400092800) on November 22, 2024. Informed consent was obtained from all participants. This study was conducted in accordance with the Declaration of Helsinki and relevant ethical guidelines and regulations.

### Study Design

**Figure 1.**
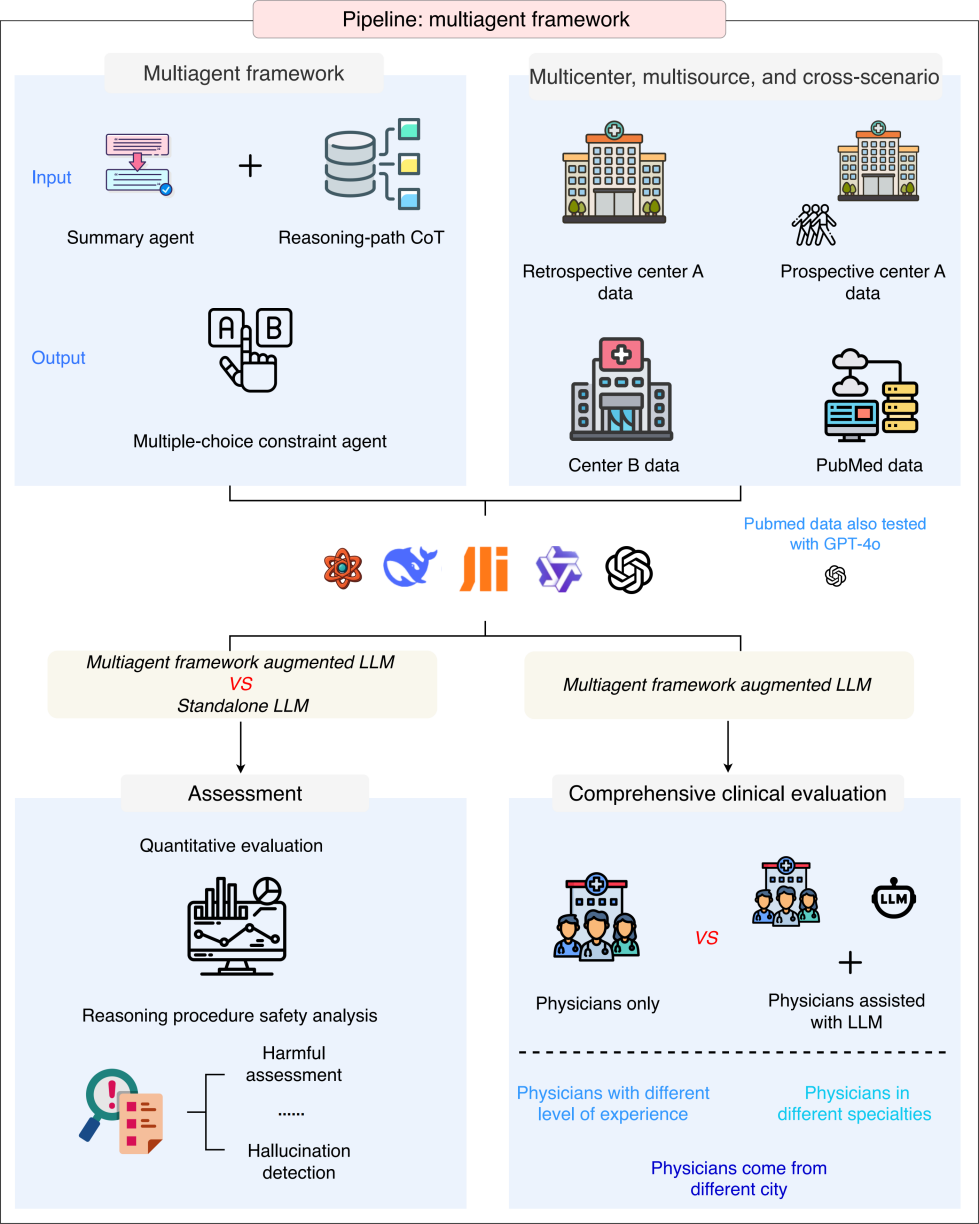
Study framework. The multiagent framework was benchmarked against standalone large language models (LLMs) and quantitatively validated across retrospective, prospective, multicenter, and PubMed datasets. Safety analyses encompassed harmful content, hallucinations, and numerical robustness, while clinical validation assessed human-AI interactions by comparing physicians’ decisions across experience levels, specialties, and geographic locations. CoT: chain of thought.

The overall study design and data sources are summarized in Figure 1. This schematic highlights the integration of multicenter retrospective and prospective real-world clinical cases with PubMed case reports, the application of the multiagent framework, and the evaluation of both model performance and human-AI interaction.

### Data Collection

We retrospectively screened clinical cases from 2 tertiary care centers: 1228 cases from center A (group A, January 2018-January 2025, tertiary grade A hospital) and 938 cases from center B (group B, May 2018-March 2025, tertiary grade B hospital). All cases were deidentified encounters of patients diagnosed with acute cerebrovascular disease. In addition, 327 stroke case reports were retrieved from PubMed between January 2024 and January 2025 (group C), which predominantly represented diagnostically challenging and clinically complex presentations. For prospective validation, 213 patients were consecutively enrolled at center A between February and May 2025 (group D). Detailed inclusion and exclusion criteria for each group are provided in the Supplementary Material Methods section and Figure S1 in Multimedia Appendix 1.

### Patient Cases

To ensure patient privacy, all personally identifiable information was removed. Each case was formatted as a single paragraph containing all or a subset of the following elements: patient age and sex, chief complaint, current symptoms, medical history (including illnesses and medications), relevant family history, physical examination findings, laboratory test results, and imaging reports.

### Evaluation of Standalone LLMs

To evaluate LLM performance in generating AIS treatment recommendations and assigning stroke subtypes according to the Trial of ORG 10172 in Acute Stroke Treatment (TOAST) classification, each model was tasked with generating a treatment recommendation and the corresponding TOAST classification conclusion. Seven models were tested: Baichuan-M1-14B [18], GPT-OSS-20B [19], Qwen2.5-32B [20], DeepSeek-R1-Distill-Qwen2.5-32B [21], GPT-OSS-120B [19], DeepSeek-R1-671B [21], and GPT-4o (OpenAI; only in group C dataset) [22] (Table S1 in Multimedia Appendix 1). Real-world clinical cases from groups A to D were used for this assessment. Outputs were produced in free-text format without predefined options, reflecting the probabilistic nature of clinical reasoning. To ensure consistent and reproducible evaluation, an automated grader agent was used to quantify accuracy across all LLMs (Supplementary Material Methods section in Multimedia Appendix 1).

All LLMs were evaluated in single-turn interactions using their default parameter configurations, without additional manual tuning. Model inference was performed with the official vLLM framework, providing an optimized environment for efficient large-scale deployment. All models were executed on a cluster of 8 NVIDIA H20-141GB graphics processing units (GPUs) using the official Docker release. For version control, vLLM v0.8.4 was applied to all models except GPT-OSS-20B and GPT-OSS-120B, which were deployed under v0.10.1, thereby ensuring reproducibility and transparency across experiments.

### The Multiagent Framework

#### Overview

In addition to standalone evaluations, we implemented a multiagent framework to examine whether structured reasoning and constrained outputs could enhance model performance in AIS-specific tasks. The framework integrates three components: (1) a workflow-oriented summary agent that extracts disease-relevant evidence from lengthy clinical narratives, (2) a guideline-concordant reasoning-path chain of thought (Short-CoT) Agent that enforces structured diagnostic steps, and (3) a clinically inspired multiple-choice constraint agent that standardizes outputs within evidence-based decision boundaries.

#### Summarization Agent

To mitigate performance degradation caused by lengthy case tokens and to ensure the extraction of salient details, a workflow-oriented summary agent was implemented. Using DeepSeek-R1 with few-shot prompting, this agent generated structured case summaries for downstream reasoning.

#### Short-CoT Agent

The Short-CoT module was designed as a concise sequence of 4 critical diagnostic steps, mirroring clinical decision trees. It was developed collaboratively with neurologists, interventional radiologists, and emergency physicians by restructuring existing clinical guidelines (refer to the prompt provided in Supplementary Material Methods in Multimedia Appendix 1).

#### Multiple-Choice Constraint Agent

For clinically inspired final decision-making, models augmented by the multiagent framework were evaluated using 4 predefined categories as answer options: *thrombolysis, mechanical thrombectomy, standard medical therapy, and non-AIS or nonstroke conditions*. This constraint not only improved consistency but also aligned model outputs with clinically interpretable categories.

### Alternative Framework Compositions

We also evaluated 3 alternative framework compositions: F1 included the Short-CoT agent and the multiple-choice constraint agent but omitted the summary agent; F2 included the guideline-derived long CoT (Long-CoT) agent and the multiple-choice constraint agent but omitted the summary agent; F3 included the summary agent and the Long-CoT agent combined with the multiple-choice constraint agent. This part used a Long-CoT agent to augment the selected LLM decision-making. The Long-CoT involved filtering and restructuring clinical guidelines into a structured decision-making pathway for AIS (refer to the prompt provided in Supplementary Material Methods in Multimedia Appendix 1) [23].

### Model-Level Performance and Output Safety Assessment

Ground truth for model-level evaluation was defined as the actual treatment decision and corresponding TOAST classification documented in the clinical record, with independent verification for guideline concordance performed by 2 senior stroke clinicians: a neurologist with >10 years of experience and a neurointerventional physician with >15 years of experience. Cases with disagreement or ambiguity regarding guideline concordance were jointly re-evaluated, and eligibility for inclusion was determined by consensus.

The model-level primary outcome was the overall therapeutic and TOAST diagnostic accuracy of LLMs. Accuracy was the case-level proportion correct; TOAST F1 was macroaveraged one-vs-rest F1 across classes, and diagnostic F1 was computed on binary correctness (1/0). Model-level secondary outcomes encompassed safety-related assessments, including instruction adherence, structured harmfulness assessment, and other exploratory safety metrics. For each case, the presence of any omission or hallucination was counted as one event (yes or no), and event rates were computed as events per case (events/total cases) [24,25].

### Comprehensive Multicenter, Multisource, and Cross-Scenario Evaluation

#### Validation Using PubMed Case Reports and External Cohorts

To clinically validate the framework, we analyzed real-world cases from group B using 6 open-source LLMs. To further test generalizability across model families, we also evaluated 6 open-source LLMs and an additional closed-source LLM in group C (PubMed case reports). The PubMed case report dataset is publicly accessible and predominantly comprises diagnostically challenging cases. This dataset therefore provided a more stringent test of the generalizability of the multiagent framework across patients with varying levels of difficulty and complexity.

#### Prospective Clinical Validation With Multilevel and Multispecialty Physicians

To evaluate the clinical impact of LLM assistance, we conducted a prospective, blinded physician study at center A (approval 2024-KY-203) between February and May 2025. Twelve physicians with heterogeneous AIS expertise were recruited across 4 Chinese cities or provinces (Hunan, Guangdong, Jilin, and Shanghai), comprising junior (n=5), senior (n=5), and expert strata (n=2) and including stroke specialists and nonspecialists. Consecutive eligible patients were enrolled and randomized at enrollment to an AI-assisted arm (with LLM support) or a standard review arm (without LLM support). Each case was evaluated only once by a single assigned physician.

In the AI-assisted arm, the best-performing model from the retrospective evaluation (DeepSeek-R1) generated treatment recommendations, TOAST classifications, and structured reasoning. These model outputs were provided to physicians together with the routinely available clinical materials for each assigned case. Physicians additionally provided a 5-point Likert rating of the perceived effectiveness of the AI-assisted reasoning (with higher scores indicating greater perceived usefulness). In the standard review arm, physicians reviewed identical case materials without any model output or additional computer-based decision support beyond routine hospital systems.

The reference standard was the final multidisciplinary team decision. Additional design details, including sample-size estimation and allocation procedures, are reported in the Supplementary Material Methods in Multimedia Appendix 1.

### Statistical Analysis

All analyses were performed in Python (version 3.10; Python Software Foundation). Statistical significance was set at a 2-sided *P*<.05. Binary and continuous variables were summarized descriptively. Accuracy differences between standalone and LLMs augmented by the multiagent framework were tested with the McNemar test, and *F*_1_-score differences were estimated by bootstrap resampling (1000 iterations) with 95% CIs. Paired ordinal outcomes were compared using the Wilcoxon signed-rank test. The ablation study compared the full multiagent framework with predefined variants that removed or substituted key modules (summary agent, guideline-derived Long-CoT vs Short-CoT, and free-form vs constrained multiple-choice outputs) [26]. We fitted physician-case–level binomial-logit generalized linear mixed-effects models (GLMMs), with decision correctness as the binary outcome, and LLM support as the main fixed effect for treatment decision and TOAST classification [27]. Event rates—including omission and hallucination frequencies—and differences between AI-assisted and nonassisted arms were analyzed using chi-square or Fisher exact tests, as appropriate. Outcome metrics and definitions are summarized in Table S2 in Multimedia Appendix 1.

## Results

### Patient Characteristics

At center A, 1228 cases were screened retrospectively, and 1055 (85.91%) were included in group A. At center B, 938 were screened, and 721 (76.87%) were included in group B. In addition, 213 prospective cases were screened at center A between February and May 2025, of which 161 (75.59%) were included in group D. Of 327 PubMed case reports retrieved, 144 (44.04%) met the inclusion criteria and were included in group C. We finally analyzed 1937 clinical cases (mean age 68.0, SD 13.6 years; n=761, 39.29% women) between January 2018 and May 2025, and 144 PubMed case reports identified from January 2024 to January 2025 (Table 1).

**Table 1.** Characteristics of enrolled patients.

Variables	Group A (center A; n=1055)	Group B (center B; n=721)	Group C (PubMed cases; n=144)	Group D (center A; n=161)
Age (y), mean (SD)	69.86 (13.26)	66.55 (11.76)	56.46 (18.73)	72.32 (12.22)
Male, n (%)	665 (63.03)	485 (67.27)	71^a^ (50)	97 (60.25)
Female, n (%)	390 (36.97)	236 (32.73)	71^a^ (50)	64 (39.75)
Disease categories, n (%)				
AIS^b^	998 (94.6)	610 (84.6)	127 (88.19)	159 (98.76)
Cerebral hemorrhage	26 (2.46)	64 (8.88)	1 (0.69)	1 (0.62)
Epilepsy	8 (0.76)	18 (2.5)	1 (0.69)	0 (0)
Arterial aneurysm	4 (0.38)	3 (0.42)	0 (0)	1 (0.62)
Transient ischemic attack	9 (0.85)	22 (3.05)	4 (2.78)	0 (0)
Other non-AIS diseases	10 (0.95)	4 (0.55)	11 (7.64)	0 (0)
AIS treatment n (%)^c^				
Thrombolysis	135 (13.53)	135 (22.13)	17 (13.39)	20 (12.58)
Endovascular thrombectomy^d^	297 (29.76)	125 (20.49)	44 (34.56)	27 (16.98)
Standard medical management	565 (56.61)	350 (57.38)	66 (51.97)	112 (70.44)
TOAST^e^ diagnosis of AIS n (%)^c^				
Large artery atherosclerosis	760 (76.15)	494 (80.98)	41 (32.28)	99 (62.26)
Cardioembolism	98 (9.82)	40 (6.56)	28 (22.04)	21 (13.21)
Small vessel occlusion	95 (9.52)	37 (6.07)	6 (4.7)	37 (23.27)
Other causes	26 (2.61)	15 (2.46)	45 (35.43)	1 (0.63)
Cryptogenic	18 (1.80)	24 (3.93)	5 (3.94)	1 (0.63)
NIHSS^f^, mean (SD)	8.3 (9.1)	6.3 (7.1)	6.9 (8.3)	7.1 (8.3)
Time from symptom onset (min), median (IQR)	360.0 (180-1440)	360.0 (150-1435)	660.0 (210-1440)	360.0 (210-960)

a Two cases had missing sex information.

b AIS: acute ischemic stroke.

c Percentages for AIS treatment and TOAST diagnosis were calculated using the number of confirmed AIS cases in each group as the denominator (Group A: n=998; Group B: n=610; Group C: n=127; Group D: n=159).

d Including bridging therapy (thrombolysis and endovascular thrombectomy).

e TOAST: Trial of ORG 10172 in Acute Stroke Treatment.

f NIHSS: National Institutes of Health Stroke Scale.

### Baseline Performance Disparities Among Standalone LLMs

**Figure 2.**
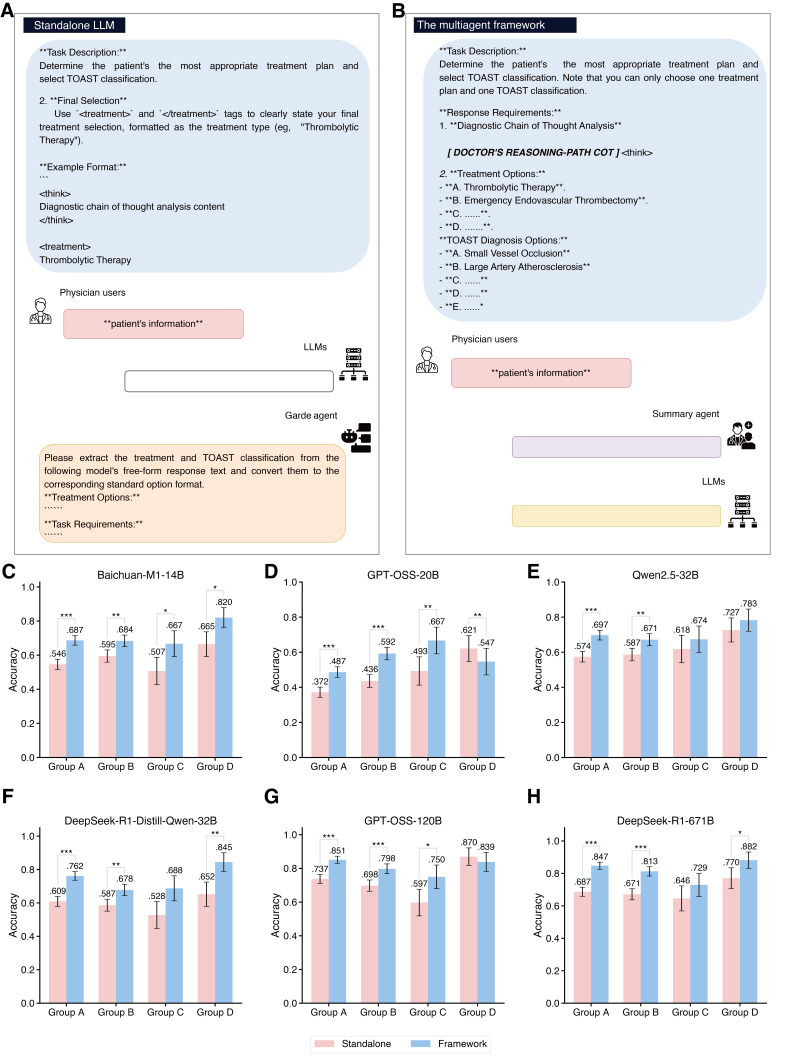
Simplified system architecture and performance of standalone vs framework-augmented large language models (LLMs). (A) and (B) Simplified system architecture: constrained inputs use <think>, <treatment>, and <diagnosis> tags. (C-H) Accuracy of acute ischemic stroke (AIS) treatment recommendation for 6 LLMs (Baichuan-M1-14B, GPT-OSS-20B, Qwen2.5-32B, DeepSeek-R1-Distill-Qwen-32B, GPT-OSS-120B, and DeepSeek-R1-671B) across groups A-C; paired bars compare standalone with multiagent, showing consistent gains. In group C, GPT-4o gained accuracy in treatment recommendation (+14.9%, 0.750 vs 0.653). TOAST: Trial of ORG 10172 in Acute Stroke Treatment. *P<.05, **P<.01, ***P<.001.

Figure 2A and 2B and Figure S2 in Multimedia Appendix 1 illustrate the simplified system architecture for the standalone LLM and the LLMs augmented by the multiagent framework. Performance varied markedly by model size. Standalone larger-scale LLMs consistently outperformed smaller ones in both treatment recommendation and TOAST classification (Table 2). Additionally, the multiagent framework consistently improved macroaveraged sensitivity and specificity across all evaluated models (Table S3 in Multimedia Appendix 1). For example, across the pooled cohort, GPT-OSS-120B achieved 0.724 accuracy for treatment recommendation, compared with 0.570 for Baichuan-M1-14B and 0.464 for GPT-OSS-20B (all adjusted *P*<.0001; Table 2). Performance trends were consistent across individual subgroups (Table S4-S7 in Multimedia Appendix 1). Similar gaps were observed for TOAST classification, with DeepSeek-R1 surpassing all smaller models. These results establish model size as a key determinant of baseline performance.

**Table 2.** Accuracy of large language models (LLMs) for treatment recommendation and Trial of ORG 10172 in Acute Stroke Treatment (TOAST) classification in the pooled group.

Models and accuracy^a^	Standalone LLM, accuracy	Multiagent framework, accuracy	*P* value
Baichuan-M1-14B			
Treatment	0.570 (0.547-0.591)	0.695 (0.675-0.715)	<.001
TOAST	0.623 (0.602-0.645)	0.657 (0.637-0.677)	.10
GPT-OSS-20B			
Treatment	0.464 (0.441-0.486)	0.541 (0.519-0.56)	<.001
TOAST	0.530 (0.507-0.553)	0.623 (0.602-0.645)	<.001
Qwen2.5-32B			
Treatment	0.593 (0.574-0.615)	0.693 (0.674-0.713)	<.001
TOAST	0.645 (0.622-0.666)	0.706 (0.686-0.726)	<.001
DeepSeek-R1-Distill-Qwen-32B			
Treatment	0.599 (0.577-0.620)	0.734 (0.716-0.754)	<.001
TOAST	0.643 (0.622-0.663)	0.689 (0.669-0.708)	<.001
GPT-OSS-120B			
Treatment	0.724 (0.706-0.744)	0.825 (0.808-0.840)	<.001
TOAST	0.690 (0.668-0.710)	0.711 (0.690-0.732)	<.001
DeepSeek-R1-671B			
Treatment	0.685 (0.667-0.704)	0.830 (0.813-0.847)	<.001
TOAST	0.680 (0.659-0.701)	0.758 (0.738-0.777)	<.001

a Values are presented as point estimates, with 95% confidence intervals shown in parentheses.

### Multiagent Framework-Induced Improvements Across LLMs

**Figure 3.**
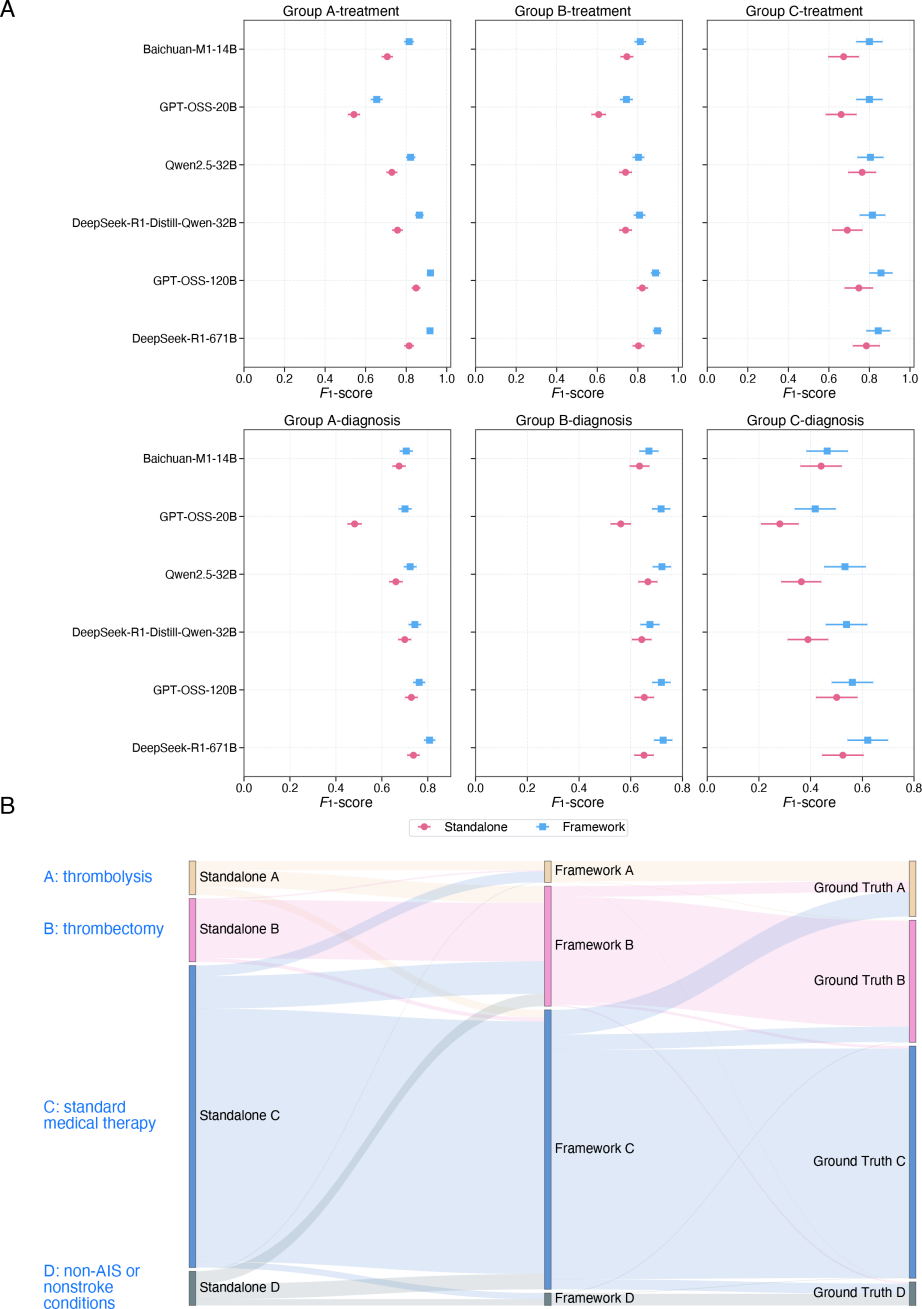
*F*_1_-score comparison of large language models (LLMs) in acute ischemic stroke (AIS) treatment recommendation and Trial of ORG 10172 in Acute Stroke Treatment (TOAST) classification (A) and treatment recommendation flows (B). (A) *F*_1_-scores are shown for AIS treatment recommendation and TOAST classification across groups A-C and 6 LLMs of increasing scale. Within each LLM, paired dots represent standalone performance (orange) and framework-augmented LLMs (blue), with error bars indicating CIs. The *F*_1_-score highlights the enhanced diagnostic reliability of the multiagent framework. (B) Sankey diagram comparing treatment recommendation flows between the standalone and the multiagent framework. Asterisks denote within-group differences between conditions, 2-sided paired tests. *P<.05, **P<.01, ***P<.001.

Augmentation with the multiagent framework substantially improved outcomes across all models (Figure 2C-2H and Figure 3A; Figure S3 in Multimedia Appendix 1), with an average improvement of 18.9% compared with standalone LLMs. In group A, Baichuan-M1-14B accuracy increased by 25.8% (*F*_1_-score +15.1%), while DeepSeek-R1-671B improved more modestly (+23.3% accuracy and *F*_1_-score +12.7%). For TOAST classification, GPT-OSS-20B accuracy rose by 39.0% (*F*_1_-score +4.9%), compared with only 2.5% (*F*_1_-score −2.1%) for GPT-OSS-120B. Notably, GPT-OSS-20B exhibited unstable outputs with fluctuating gains. Collectively, these findings show that while model size remains critical for baseline accuracy, the multiagent framework reduces size-related disparities, enabling smaller models to approach the clinical utility of their larger counterparts.

### Cross-Group and Multisource Validation

**Figure 4.**
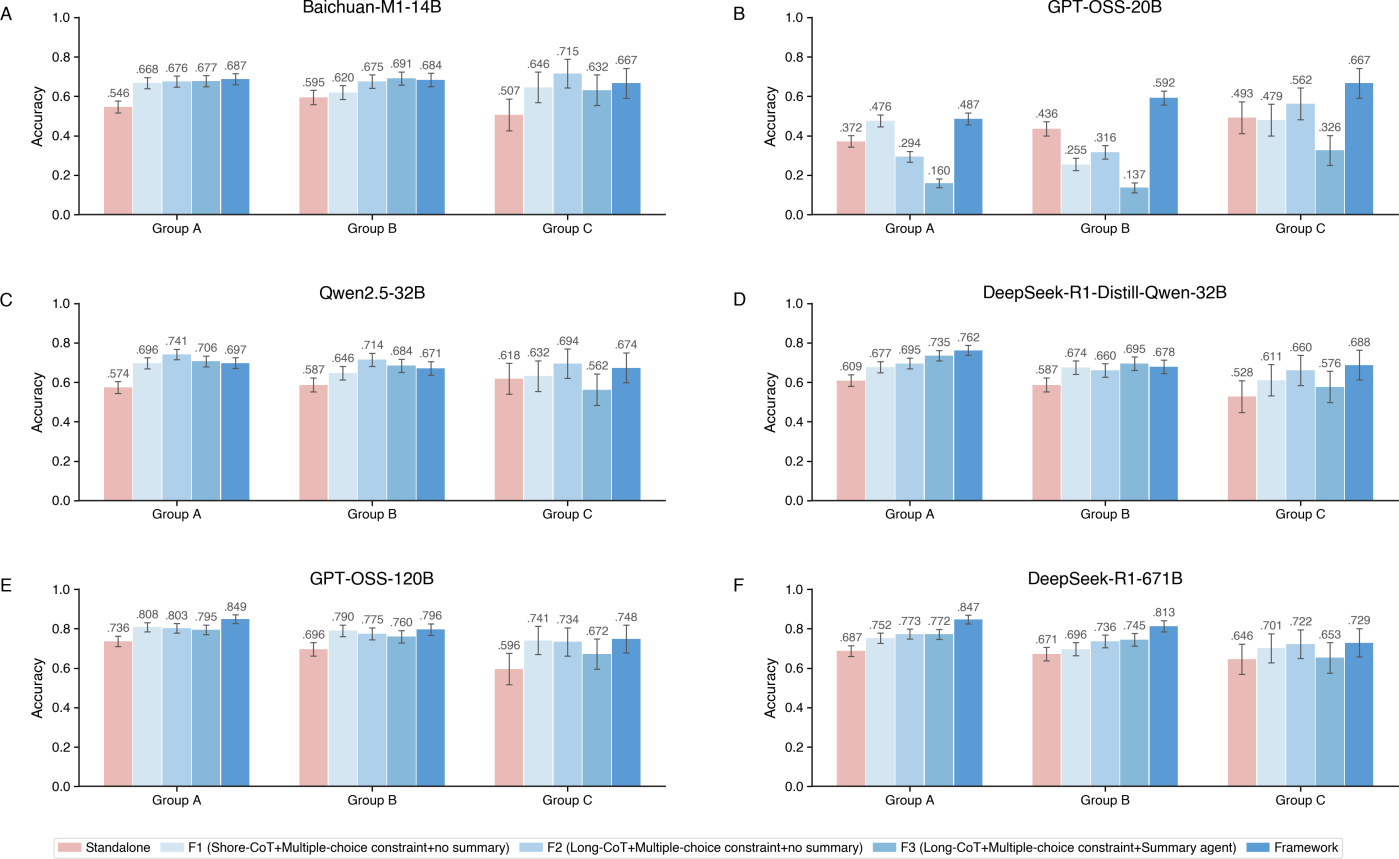
Performance of standalone large language models (LLMs), 3 alternative framework compositions, and our multiagent framework. Panels A-F show accuracy for acute ischemic stroke (AIS) treatment recommendation across groups A-C. Paired bars summarize accuracy profiles across settings, providing a direct comparison of different framework compositions*.*

Validation in groups B and C demonstrated consistent performance gains across all groups (Figure 2C-2H and Figure 3A; Tables S4-S7 in Multimedia Appendix 1). The framework-augmented DeepSeek-R1-671B achieved the highest accuracy in both treatment recommendation and TOAST classification. In group C, GPT-4o gained accuracy in treatment recommendation (+14.9%, 0.750 vs 0.653) but showed a decline in TOAST classification (−6.7%, 0.486 vs 0.521), reflecting the predominance of “other causes” and cryptogenic subtypes. The results for alternative framework compositions are shown in Figure 4. Collectively, these findings confirm that the framework provides reliable gains across heterogeneous cohorts.

### Contribution of Framework Components

Generalized linear mixed model analysis in group A confirmed that the multiagent framework was independently associated with higher accuracy in AIS treatment recommendations (odds ratio [OR] 1.72, 95% CI 1.63-1.81; *P*<.001) and increased selection of appropriate reperfusion strategies (Figure 3B).

In the ablation study (average-only aggregation), treatment recommendation performance improved when using a multiple-choice constraint compared with free-form answer formats (constrained vs free-form: +0.038), and CoT prompting outperformed direct prompting (long vs direct: +0.052; short vs direct: +0.063), suggesting that treatment recommendations benefit from structured answer formats and explicit reasoning scaffolds. Summarization yielded only a modest average gain for treatment recommendation (summary vs no summary: +0.006). In contrast, diagnosis benefited more from summarization (+0.041) and performed better with free-form answer formats (constrained vs free-form: −0.051), motivating task-specific choices of prompting strategy and answer-format control.

### Qualifying LLM Reasoning for Safe Clinical Deployment

**Figure 5.**
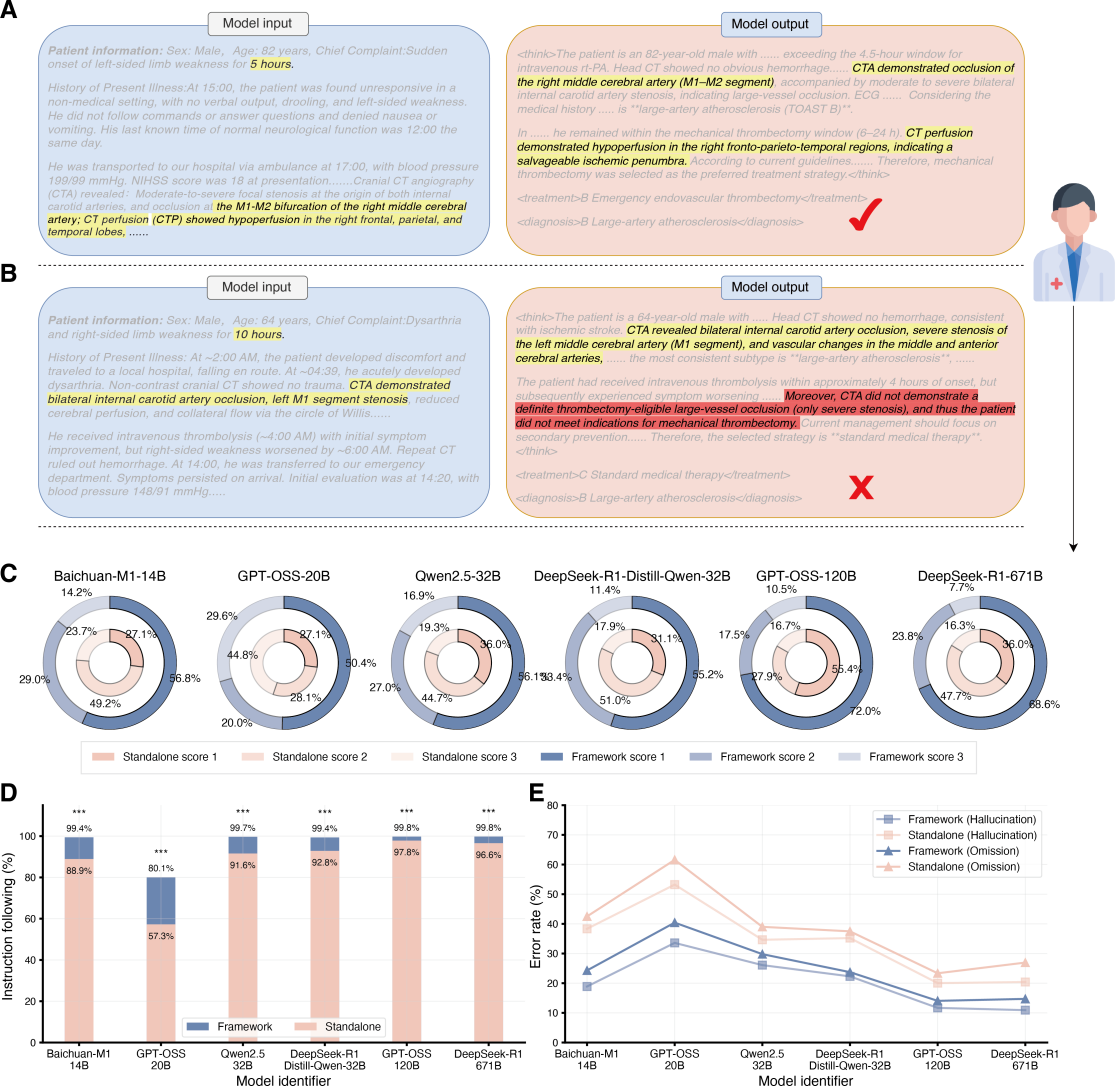
Case examples and safety profile of large language model (LLM) outputs. (A-B), Representative cases. (A) Correct recommendation with faithful reasoning and no hallucination or omission. (B) Incorrect recommendation: relevant details were identified, but hallucinated content led to an erroneous conclusion. (C-E) Safety metrics across 6 LLMs. (C) Harmfulness ratings on a 3-point scale (1=not harmful; 3=highly harmful) shown as a concentric doughnut plot with overlaid points. (D) Instruction-following compliance is shown as a bar plot of adherence proportions. (E) Incidence rates of hallucination and omission are shown as line plots. *P<.05, **P<.01, ***P<.001.

Beyond accuracy, we visualized step-by-step reasoning traces for representative success and failure cases to characterize failure modes and deployment-relevant clinical safety risks (Figure 5A and 5B). Because accuracy alone provides an incomplete view of clinical applicability, we further assessed output safety. Compared with the standalone LLM, the multiagent framework achieved higher mean clinical safety scores (4.01, SD 0.35 vs 3.70, SD 0.43) and lower mean hallucination (20.6% vs 33.6%) and omission rates (24.5% vs 38.5%; Figure 5C-5E; Table S8 in Multimedia Appendix 1). Other exploratory safety metrics are shown in Table S9 in Multimedia Appendix 1. These findings demonstrate that structured reasoning may improve reliability and mitigate the risk of unsafe content entering clinical workflows.

### LLM Support Benefits Less-Experienced Physicians

**Figure 6.**
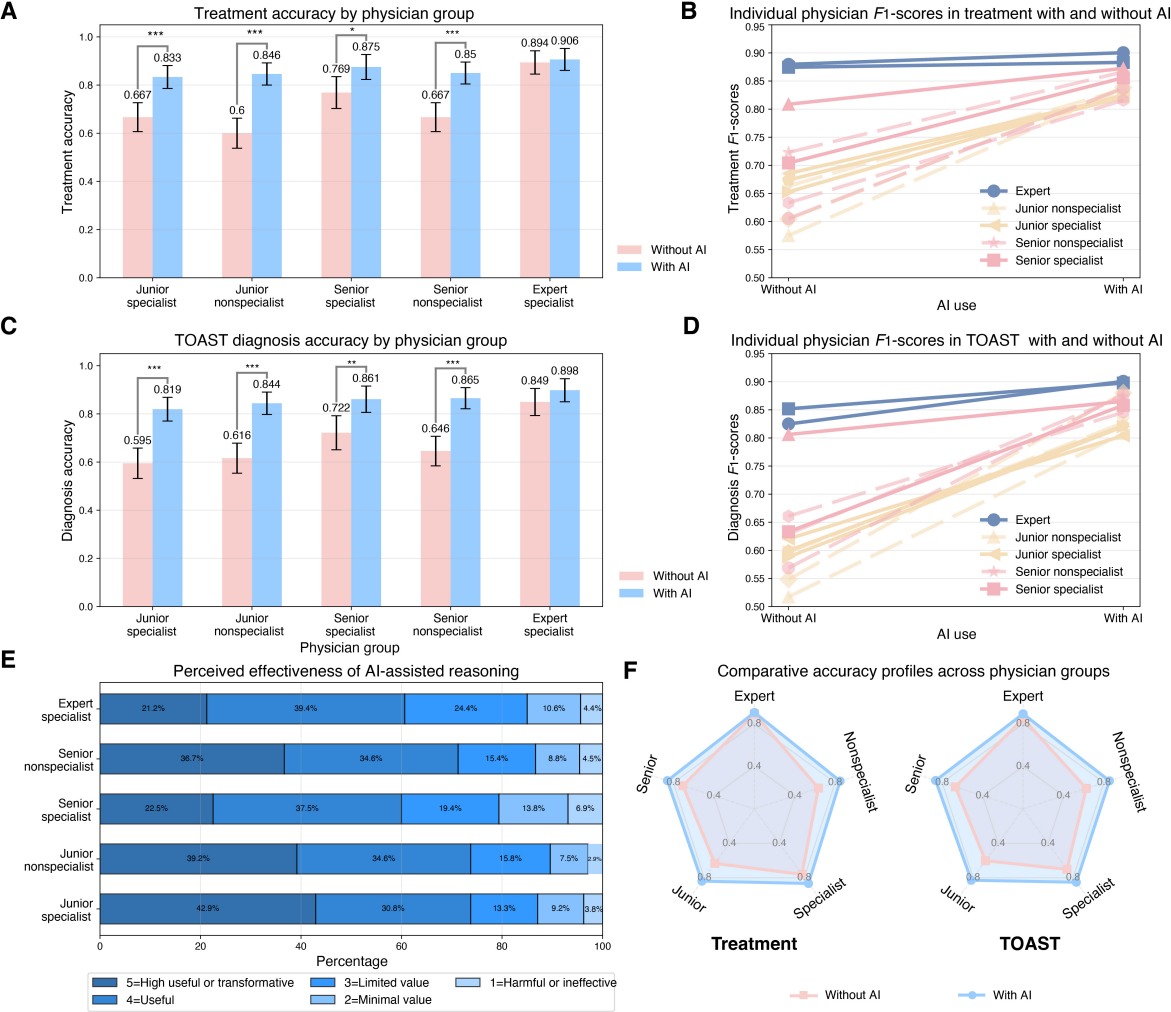
Human-AI evaluation across physician groups. (A) Treatment recommendation accuracy without AI (pink) and with AI (blue); bars show means with 95% CIs. (B) Individual physicians’ treatment *F*_1_-scores; lines connect the same physician from “without AI” to “with AI.” (C) Trial of ORG 10172 in Acute Stroke Treatment (TOAST) classification accuracy by physician group, as in panel A. (D) Individual physicians’ TOAST *F*_1_-scores, as in panel B. (E) Perceived effectiveness of AI-assisted reasoning on a 5-point Likert scale (5=highly useful or transformative; 1=harmful or ineffective), stratified by physician group. (F) Radar plots summarizing accuracy profiles for treatment recommendation (left) and TOAST classification (right), with and without AI assistance. Physician groups are stratified by seniority (junior vs senior, including both specialists and nonspecialists) and by specialty background (specialist vs nonspecialist, including both junior and senior physicians). *P<.05, **P<.01, ***P<.001.

Integration of LLM support substantially enhanced physician performance, with the most pronounced gains observed for less-experienced physicians in treatment decisions (0.667 to 0.833 in junior specialists; 0.600 to 0.846 in nonspecialists). On the basis of physician-case decision-level observations (1 observation per physician per case), we fitted a binomial-logit GLMM with decision correctness as the binary outcome, LLM support, physician experience, and specialty as fixed effects, and a random intercept for case and physician. After adjustment, LLM support was associated with higher odds of correct treatment decisions (OR 2.86, 95% CI 2.27‐3.60; *P*<.0001) and correct TOAST classification (OR 3.63, 95% CI 2.85‐4.64; *P*<.0001; Figure S4 in Multimedia Appendix 1). The estimated association was strongest among junior and nonspecialist physicians. For treatment decisions, the ORs were 3.96 (95% CI 2.64-5.96) for junior nonspecialists and 3.22 (95% CI 2.13-4.87) for senior nonspecialists; for TOAST classification, the corresponding ORs were 4.66 (95% CI 3.01-7.22) and 4.27 (95% CI 2.74-6.66). Expert specialists showed positive but nonsignificant estimates, consistent with a ceiling effect at higher baseline performance levels (*P*<.001). Physicians further rated the assistance positively, with a mean score of 3.849 out of 5. Improvements were more modest in senior and specialist physicians (Figure 6).

## Discussion

This study advances the clinical translation of LLMs for AIS decision support by moving beyond benchmark-style evaluation to a multicenter, multisource assessment across retrospective, prospective, and literature-derived cases. First, the structured multiagent framework improved guideline-concordant therapy selection while producing more reliable and clinically accountable outputs, with lower hallucination and omission rates. Second, in a human-AI interaction experiment involving physicians of varying seniority and specialty backgrounds, the framework improved physician decision accuracy, with the largest gains observed among junior and nonspecialist physicians. Collectively, these findings suggest that LLM-integrated systems can enhance both intrinsic decision reliability and downstream clinical performance, offering a practical means to improve consistency, safety, and equity in AIS care when access to stroke expertise is uneven.

Global inequities in stroke care remain profound, with low- and middle-income countries constrained by limited resources and specialist expertise, while high-income countries face overcrowded emergency services and workforce pressures. Existing evaluations of LLMs have been largely confined to simplified benchmark tasks that fail to capture the real-world complexity of disease management [16,20,21]. To address this gap, we applied LLMs to real-world clinical and literature-derived cases, thereby simulating authentic clinical scenarios and reflecting heterogeneous contexts. Performance declined in complex settings, partly due to limitations in handling long token inputs [28,29]. To address this challenge, our multiagent framework incorporated a custom-designed summarization agent that extracted salient features from clinical narratives, improving accuracy across diverse scenarios. Augmented LLMs achieved accuracies ranging from 0.541 to 0.830 across model sizes—a level comparable to question-and-answer–style or simulated patient scenarios [13,30], and consistently higher than standalone LLMs.

This study represents an initial step toward translating LLM-assisted decision support for AIS into clinical practice, with a focus on reducing variability in care that arises from uneven clinical expertise. Prior work suggests that CoT prompting and fixed-answer formats can partially address these limitations [31,32]. Our multiagent framework introduces a workflow-oriented, guideline-concordant structure that improves decision accuracy while maintaining clinically consistent recommendations, supporting feasibility for future clinical integration. Unlike approaches that rely on ever-larger proprietary models [33], our results show that workflow-inspired structured design improvements can yield substantial gains without increasing model scale, potentially lowering adoption barriers and supporting broader access to specialist-level decision support.

Our evaluation offers one of the most comprehensive simulations of clinical practice to date, establishing a foundation for the deployment of LLMs in AIS workflows. While the promise is substantial, deployment at scale carries risks of unintended harmful consequences [34,35]. By integrating data from multicenter, multitier hospitals, retrospective and prospective real-world cases, literature-derived high-difficulty cases, and human-AI interactions across physicians of different levels and specialties, our evaluation captured the heterogeneity and complexity of AIS care. Notably, LLMs provided the most benefit to less-experienced physicians, narrowing expertise gaps across experience, specialty, and geography, and aligning with prior reports of near expert-level performance [36,37]. In alignment with the World Stroke Organization’s Global Stroke Declaration, which underscores that quality stroke care should be universal [2,38], our results advance the case for AI-enabled strategies to promote equity in global stroke systems.

This study systematically assessed safety, a critical prerequisite for clinical deployment. Because LLMs predict the next token without verifying evidence, they remain prone to hallucinations that can erode trust and generate harmful or misleading recommendations [39,40]. In our evaluation, hallucinations and omissions were not eliminated but occurred at relatively low frequencies, with the multiagent DeepSeek-R1-671B achieving a hallucination rate of 10.9%, an omission rate of 14.7%, and an overall clinical safety score of 4.36 out of 5. These findings indicate that while safety concerns remain, the error rates are within a range that may be acceptable for decision support use, supporting the feasibility of cautious clinical integration [41]. The multiagent-augmented LLMs further demonstrated stronger instruction adherence, lower hallucination and omission rates, and more reliable structured outputs, thereby addressing a key barrier to real-world deployment.

Our results suggest that structured multiagent LLM frameworks may improve guideline-concordant AIS decision support, output safety, and auditability, but the remaining safety-critical errors highlight the need for caution before clinical deployment. Such systems should be used only as human-in-the-loop decision-support tools, with final treatment decisions remaining under physician responsibility. Future workflow-based studies should prospectively test safeguards, including uncertainty signaling, guideline and local-protocol alignment, contraindication review, deferral under missing or ambiguous information, high-risk warnings, and complete logging of model inputs, outputs, and reasoning traces.

This study has several limitations. First, the framework was evaluated in case-based rather than real-time emergency room settings, precluding assessment of usability, clinician trust, latency, time to decision, bedside integration, patient outcomes, and uncertainty escalation. Second, the exclusion criteria may have produced a more standardized dataset than real-world emergency stroke care. By excluding cases with incomplete information, chronic-phase presentations, competing urgent conditions, or treatment refusal or discontinuation, the study may underrepresent information-limited and operationally constrained scenarios. Third, although the framework reduced omission and hallucination events, the proposed deployment guardrails were not prospectively tested in real clinical workflows, limiting conclusions about operational safety. Fourth, the automated grader used to categorize free-text standalone LLM outputs may have introduced evaluation error, particularly for ambiguous or internally inconsistent responses. Finally, rapid iteration of commercial LLMs and restricted access to proprietary systems may affect reproducibility and long-term stability. Future prospective multicenter workflow studies should evaluate the framework under more complex real-world conditions, with predefined safety guardrails, human adjudication of uncertain outputs, and patient-level outcome assessment.

In conclusion, our multicenter evaluation demonstrates that a structured, multiagent LLM framework significantly enhances guideline-concordant AIS decision-making (average accuracy gain: 18.9%) and output safety. Crucially, LLM support substantially increased the odds of correct physician decisions for both treatment (OR 2.86, 95% CI 2.27‐3.60) and TOAST classification (OR 3.63, 95% CI 2.85‐4.64), with the most pronounced benefits among less-experienced and nonspecialist clinicians. These findings highlight the framework’s potential to narrow expertise gaps and support equitable stroke care, warranting further prospective evaluations of real-world workflows and patient outcomes before clinical deployment.

## Supplementary material

10.2196/96304 Multimedia Appendix 1 Supplementary results, including study cohort flow and additional analyses.

## Abbreviations

AIS: acute ischemic stroke
CoT: chain of thought
GLMM: generalized linear mixed-effects model
GPU: graphics processing unit
LLM: large language model
Long-CoT: guideline-derived long chain of thought
OR: odds ratio
Short-CoT: reasoning-path chain of thought
TOAST: Trial of ORG 10172 in Acute Stroke Treatment
