# Supplementary material for: A Multiagent Large Language Model Framework for Emergency Treatment Recommendation in Acute Ischemic Stroke: Development and Validation Study
Source: J Med Internet Res. 2026 Jul 30;28:e96304. doi: 10.2196/96304 (PMC13423760; doi:10.2196/96304)
Supplement: Multimedia Appendix 1 [file jmir-v28-e96304-s001.docx]

**A multi-agent LLM framework toward real-world clinical decision-making support in acute ischemic stroke**

**The Supplementary Material**

1. **Supplementary Material Methods**
   1. **Patients inclusion and exclusion criteria**
   2. **Evaluation of Standalone LLMs**
   3. **The Multi-agent Framework Augmented LLM**

1.3.1 Reasoning-Path CoT Agent

1.3.2 Alternative Framework Compositions

- 1. **Prospective physician study**

1.4.1 Study design and oversight

1.4.2 Participants

1.4.3 Case enrollment and relationship to retrospective datasets

1.4.4 Intervention, control, and workflow

1.4.5 Reference standard and outcomes

1.4.6 Sample size estimation

1.4.7 Statistical Analysis

**2. Supplementary Results**

Supplementary Figure S1. Participant flow through the study.

Supplementary Figure S2. Visualization of interaction interfaces for standalone LLM and multi-agent Framework augmented LLM.

Supplementary Material Figure S3. Multi-agent framework enhances accuracy of LLMs in TOAST classification.

Supplementary Figure S4. Forest plot of adjusted odds ratios for the association between LLM support and physician accuracy in the human–AI interaction experiment.

Supplementary Table S1. The detailed information on the six LLMs used in this study.

Supplementary Table S2. Outcome Metrics and Definitions.

Supplementary Table S3. Performance of LLMs in treatment recommendation and TOAST classification in all groups.

Supplementary Table S4. Performance of LLMs in treatment recommendation and TOAST classification in Group A.

Supplementary Table S5. Performance of LLMs in Treatment recommendation and TOAST classification in Group B.

Supplementary Table S6. Performance of LLMs in treatment recommendation and TOAST classification in Group C.

Supplementary Table S7. Performance of LLMs in treatment recommendation and TOAST classification in Group D.

Supplementary Table S8. Clinical safety indicators of LLMs during preliminary deployment.

Supplementary Table S9. Error-mode analysis of incorrect recommendations generated by the DeepSeek-R1 model during preliminary deployment.

1. **Supplementary Material Methods**
   1. **Patients inclusion and exclusion criteria**

The inclusion criteria were: (1) patients aged ≥ 18 years-old who were clinically suspected of having acute cerebrovascular disease; (2) evaluation and management undertaken in participating hospitals, with neuroimaging performed on admission. The exclusion criteria were: (1) refusal or discontinuation of treatment (e.g. due to financial constraints, perceived risks, or transfer to another facility), or inability to provide informed consent for standardized, guideline-concordant therapy; (2) incomplete clinical information (e.g. missing chief complaint, auxiliary examinations); (3) urgent conditions requiring interventions more immediate than stroke; (4) non-acute cerebrovascular admissions in which stroke was identified only during hospitalization; and (5) patients in the chronic phase of cerebrovascular disease. Guideline adherence of all patient treatments was assessed based on expert evaluation.

**1.2 Evaluation of Standalone LLMs**

To ensure consistent and reproducible evaluation, an automated grader agent was employed to quantify accuracy across all LLMs. The grader agent, instantiated using DeepSeek-R1, operated in two sequential steps. First, it extracted and categorized each output into one of three formats: (1) single treatment recommendation/diagnosis, (2) multiple decisions, or (3) no decision. For multiple recommendations, a prespecified Top-1 rule was used (first explicit recommendation); responses without an explicit recommendation were scored as incorrect. Second, extracted responses were compared against ground-truth references, with treatment mapped to four predefined categories: *Thrombolysis, Mechanical Thrombectomy, Standard Medical Therapy, and Non-Acute Ischemic Stroke or Non-Stroke Conditions*. This standardized pipeline minimized subjectivity and ensured consistency across experiments.

**1.3 The Multi-agent Framework**

**1.3.1 Reasoning-Path CoT Agent**

The Reasoning-Path CoT module was designed as a concise clinician-derived reasoning scaffold rather than a direct injection of full guideline text. Its structure was informed by key decision points from contemporary AIS treatment guidelines, including the AHA/ASA guideline for early management of AIS, and by routine clinical reasoning used in emergency stroke assessment. The guideline-concordant doctor’s Reasoning-Path CoT module’s prompt was:

**The diagnostic and treatment process for AIS should include the following key steps:

1. Is it a stroke? Exclude non-vascular diseases.

2. Is it an ischemic stroke? Perform brain CT/MRI to exclude hemorrhagic stroke.

3. Stroke severity? Use neurological function assessment scales to evaluate the degree of neurological deficit.

4. Can thrombolytic therapy be performed? Is endovascular treatment necessary? Check indications and contraindications.

5. Evaluate for clinical situations requiring neurosurgical consultation, such as malignant middle cerebral artery infarction or large cerebellar infarction compressing the brainstem.

6. Combine medical history, laboratory data, brain lesions, and vascular lesions to perform TOAST classification: Small vessel occlusion (lacunar infarct ≤1.5 cm), large artery atherosclerosis (cortical infarct + stenosis ≥50%), cardioembolism (embolic pattern + cardiac source), other causes (vasculitis, dissection, hypercoagulability), cryptogenic (undetermined etiology).

7. Final treatment plan determination: Thrombolytic therapy (intravenous thrombolysis within the time window), emergency endovascular thrombectomy (large vessel occlusion within the time window), standard medical therapy (antiplatelet/anticoagulant, secondary prevention), non-acute management (supportive care for non-stroke or chronic conditions).

**1.3.2 Alternative Framework Compositions**

The long-CoT involved filtering and restructuring clinical guidelines [refer.11 12] into a structured decision-making pathway for AIS, the prompt was:

**Task Description:**

Please determine the most appropriate treatment approach and TOAST classification based on the patient's condition, strictly following the guideline diagnostic process. Note that you can only select one treatment approach and one TOAST classification.

**Guideline Diagnostic Process:**

### 1. **Hospital Admission and Initial Assessment**

├─ **Symptoms**: Suspect stroke if sudden onset of:

│ (1) Unilateral limb weakness/numbness;

│ (2) Facial numbness/deviation of the mouth corner;

│ (3) Speech impairment or comprehension difficulty;

│ (4) Conjugate gaze deviation/neglect;

│ (5) Monocular/binocular vision loss/blurring;

│ (6) Vertigo with vomiting;

│ (7) Gait instability;

│ (8) Severe atypical headache/vomiting;

│ (9) Altered consciousness/seizures.

├─ **Time of onset**: Patient-reported or witness-confirmed last known well time.

├─ **Vital signs monitoring**: Blood pressure (strictly <185/110 mmHg), heart rate, respiratory rate, SpO₂.

├─ **Medical history collection**.

└─ **Neurological assessment**: NIHSS (National Institutes of Health Stroke Scale) to evaluate stroke severity.

### 2. **CT Imaging Evaluation**

│

├─ **Intracerebral hemorrhage**

│ └─ **CTA/MRA**:

│ ├─ **No aneurysm detected**:

│ │ ├─ Immediate anticoagulation;

│ │ ├─ BP control to target within 1 hour;

│ │ └─ Neurosurgical intervention if indicated.

│ └─ **Aneurysm detected**:

│ └─ Do not enter ischemic stroke treatment protocol.

│

└─ **No hemorrhage**: Proceed to ischemic stroke protocol.

---

### 3. **<4.5 Hours (Onset-to-Treatment Time)**

│

├─ **Imaging**:

│ ├─ Non-contrast head CT: Exclude hemorrhage.

│ ├─ **Normal CT**: Consider IV thrombolysis, proceed to subsequent IV thrombolysis protocol.

│ └─ **Contraindications to CT**: Proceed with MRI.

│

├─ **IV Thrombolysis (Alteplase)**:

│ ├─ **Indications**:

│ │ ├─ Confirmed ischemic stroke;

│ │ ├─ Onset-to-arrival ≤4.5 hours;

│ │ └─ No bleeding diathesis.

│ ├─ **Contraindications**:

│ │ └─ Intracranial hemorrhage (parenchymal hemorrhage, subarachnoid hemorrhage, subdural/epidural hematoma, etc.), previous history of bleeding or tumor, severe head trauma or stroke within 3 months, major surgery within 3 months or systemic bleeding, unexplained active bleeding, definite bleeding diathesis, large aneurysm, giant aneurysm or vascular malformation, known or suspected dissecting aneurysm, coagulopathy (PT >15 seconds or INR >1.7, anticoagulant use, prolonged APTT, abnormal TT or ECT), known platelet count <100×10⁹/L, severe liver disease (such as cirrhosis or liver failure), and imaging (CT or MRI) confirmed extensive cerebral infarction (such as with cerebral edema or extensive infarction, ASPECTS score <6).

│ ├─ **Protocol**:

│ │ ├─ Alteplase 0.9 mg/kg (max 90 mg): 10% bolus + 90% infusion over 1 hour.

│ │ └─ Delay antiplatelet/anticoagulant therapy for 24 hours.

│

└─ **If large vessel occlusion is identified or thrombolysis contraindications or no symptom relief after thrombolysis or family considers thrombectomy**: Proceed to mechanical thrombectomy (Step 4).

### 4. **4.5-24 Hours (Onset-to-Treatment Time)**

│

├─ **NIHSS assessment**:

│ ├─ **NIHSS ≤5, and single-item scores such as vision, language, neglect or monoparesis ≤1, for mild stroke or non-disabling stroke**: Dual antiplatelet therapy for minor stroke.

│ └─ **NIHSS 4-26 or not available**: Continue evaluation.

│

├─ **Advanced imaging**: Complete CT examination, CTA or MRA: Confirm large vessel occlusion.

│ ├─ **Anterior circulation LVO**:

│ │ ├─ 6-16 hours: CTP/MRI mismatch (if onset time 6-16 hours, CTP assessment/MRI for presence of ischemic penumbra or functionally meaningful brain tissue: DWI/FLAIR mismatch [abnormal signal on DWI but no obvious signal change on FLAIR], infarct core/perfusion mismatch [patients with infarct core volume <70ml, severe hypoperfusion/infarct volume >1.2, ischemic penumbra volume >10 ml]).

│ │ └─ 16-24 hours: CTP assessment/MRI for presence of ischemic penumbra or functionally meaningful brain tissue: DWI/FLAIR mismatch (abnormal signal on DWI but no obvious signal change on FLAIR), infarct core/perfusion mismatch (patients with infarct core volume <51ml, severe hypoperfusion/infarct volume >1.2, ischemic penumbra volume >10 ml).

│ ├─ **Posterior circulation LVO**:

│ │ ├─ patients aged 18-80, within 12 hours: NIHSS ≥6, pc-ASPECTS ≥6.

│ │ └─ 12-24 hours: Benefit-risk assessment.

│

├─ **Mechanical Thrombectomy**:

│ ├─ **Indications**: Anterior circulation LVO within 24 hours, salvageable tissue.

│ ├─ **Contraindications**: Large core infarction, coagulopathy (PT >15 seconds or INR >1.7, platelet count <100×10⁹/L), severe renal dysfunction with limited expected clinical benefit.

│ └─ **Procedure**: Catheter-based thrombectomy ± stenting.

│

└─ **Non-eligible**: Supportive care + rehabilitation.

### 5. **>24 Hours Post-Onset**

├─ **TOAST Classification**:

│ ├─ A. Large-Artery Atherosclerosis

│ │ Criteria:

│ │ - Clinical features of cortical, cerebellar, brainstem dysfunction (e.g., aphasia, neglect, ataxia).

│ │ - Brain imaging shows infarct >1.5 cm in cortical, cerebellar, brainstem, or subcortical region.

│ │ - ≥50% stenosis or occlusion in a relevant extracranial/intracranial artery (e.g., ICA, MCA).

│ │ - Cardiac sources of embolism are excluded.

│ │ - Supportive: history of TIA in same vascular territory, carotid bruit, claudication.

│

│ ├─ B. Cardioembolism

│ │ Criteria:

│ │ - Infarct >1.5 cm in cortical or multiple arterial territories.

│ │ - No significant stenosis (<50%) in proximal large arteries.

│ │ - A cardiac source of embolism must be identified:

│ │ ▸ High-risk: atrial fibrillation, recent MI (<4 weeks), valve disease, thrombus, myxoma, etc.

│ │ ▸ Medium-risk: PFO, atrial septal aneurysm, MVP, older MI, etc.

│ │ - If only medium-risk source is found, and no other cause identified → Possible cardioembolism.

│

│ ├─ C. Small-Vessel Occlusion

│ │ Criteria:

│ │ - Classical lacunar syndrome (e.g., pure motor hemiparesis, sensory stroke).

│ │ - Lesion <1.5 cm in subcortical or brainstem region.

│ │ - No evidence of cortical dysfunction.

│ │ - No ≥50% stenosis in ipsilateral large artery.

│ │ - No cardiac source of embolism.

│ │ - Supportive: history of hypertension or diabetes.

│

│ ├─ D. Stroke of Other Determined Etiology

│ │ Criteria:

│ │ - Uncommon causes confirmed by tests: e.g., arterial dissection, vasculitis, hypercoagulable states, sickle cell disease, malignancy, etc.

│ │ - Imaging confirms acute infarct.

│ │ - Diagnostic evidence excludes atherosclerosis and cardiac sources.

│

│ └─ E. Stroke of Undetermined Etiology

│ Criteria:

│ - No cause identified despite full evaluation; OR

│ - Multiple potential causes (e.g., AF + 50% carotid stenosis); OR

│ - Incomplete evaluation with insufficient data to classify into other types.

├─ **Management**: Antiplatelet/anticoagulant therapy per etiology.

└─ **Secondary prevention**: Statins, BP control, lifestyle modification.

### 6. **Monitoring & Supportive Care**

├─ Continuous neurological/vital sign monitoring.

├─ Complication management (hypertension, hyperglycemia).

├─ Multidisciplinary rehabilitation.

└─ Secondary prevention protocols.

**1.4 Prospective physician study**

**1.4.1 Study design and oversight**

A prospective physician study was conducted at Center A between February and May 2025 (ethics approval no. 2024-KY-203) to quantify the clinical impact of LLM assistance on time-critical AIS emergency decision-making. All participating physicians were blinded to the multidisciplinary team (MDT) reference decisions at the time of case review, and study procedures (case packaging, randomization, and outcome definitions) were prespecified.

**1.4.2 Participants**

Twelve physicians with heterogeneous AIS expertise were recruited across four Chinese cities/provinces (Hunan, Guangdong, Jilin, and Shanghai) and stratified by clinical seniority and specialty background. The cohort included 2 junior oncologists (junior non-specialists) and 3 junior interventional neurologists (junior stroke specialists) with <3 years of practice; 3 general practitioners (senior non-specialists) and 2 senior interventional neurologists (senior stroke specialists) with ~5 years of practice; and 2 expert interventional neurologists (>10 years of practice).

**1.4.3 Case enrollment and relationship to retrospective datasets**

Eligible patients were prospectively enrolled consecutively during the study period according to the prespecified inclusion and exclusion criteria. This prospective cohort was independent of the retrospective datasets used for offline evaluation; in particular, it did not include all patients from Group D.

**1.4.4 Intervention, control, and workflow**

At enrollment, patients were randomized 1:1 to an AI-assisted arm (With AI) or a standard review arm (Without AI). Cases were prospectively collected by on-call clinicians and uploaded to a shared secure web portal, from which case packages were sequentially and randomly allocated to participating physicians for independent interpretation. In the AI-assisted arm, the model integrated into the framework was DeepSeek-R1, selected a priori as the best-performing model based on retrospective evaluation (Group A). The AI package contained the model-generated treatment recommendation, TOAST classification, and structured reasoning output, presented alongside each patient’s admission history and the same clinical materials available for routine review. In the standard review arm, physicians reviewed identical case materials without any LLM output and without additional computer-based decision support tools beyond routine hospital information systems (e.g., standard PACS/EHR access).

**1.4.5 Reference standard and outcomes**

The reference standard for both treatment decisions and TOAST subtyping was the final MDT adjudication for each patient (guideline-informed multidisciplinary decision), rather than the raw treatment delivered. Physician recommendations (with or without AI assistance) were compared against this MDT reference standard.

**1.4.6 Sample size estimation**

Sample size planning targeted the hypothesis that LLM assistance would improve treatment decision accuracy among less-experienced physicians from an assumed baseline of 0.60 (Without AI) to 0.82 (With AI), approximating the performance of senior physicians observed in preliminary analyses. Using a two-sided α=0.05 and 80% power for a two-sample comparison of proportions with equal allocation, the required sample size was calculated as:


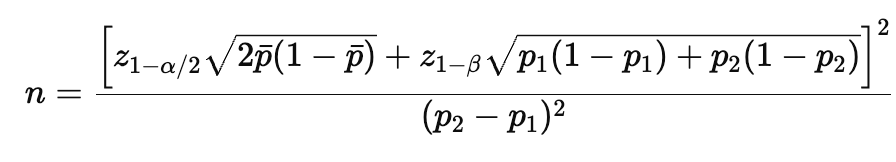


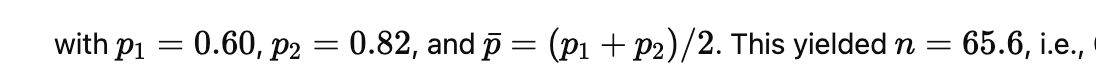
66 cases per arm (132 total). To account for exclusions and incomplete physician assessments, we prespecified an inflation margin (~10%), targeting approximately 72 cases per arm (~144 total). For reference, achieving 90% power under the same assumptions would require ~87 cases per arm.

**1.4.7 Statistical Analysis**

For the physician evaluation study, each observation corresponded to a physician-case-condition decision, with the binary outcome defined as whether the physician’s treatment recommendation matched the clinical reference standard. The primary fixed effect was decision condition, defined as without versus with LLM support. Physician seniority was included as an additional fixed effect to account for differences in clinical experience. To account for repeated measures and the crossed structure of the data, we fitted a binomial generalized linear mixed-effects model with random intercepts for physician and case. This model estimated the independent association between LLM support and physician decision accuracy while accounting for correlation among decisions made by the same physician and among repeated evaluations of the same case.

1. **Supplementary Results**

**Supplementary Figures**


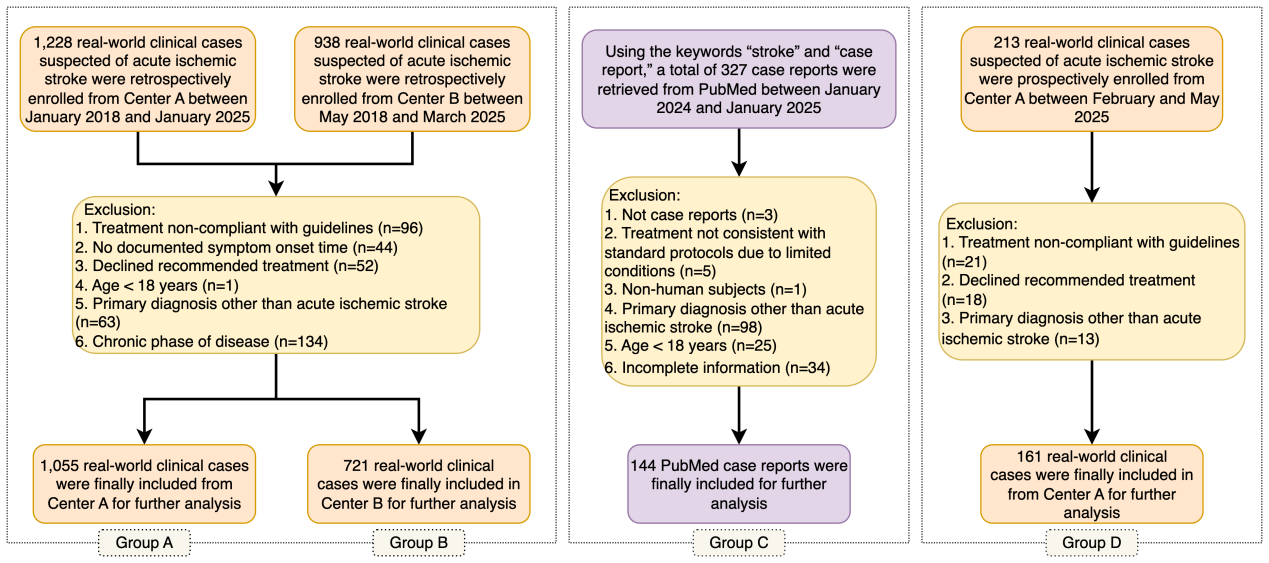


**Supplementary Figure S1. Participant flow through the study.** Flow diagram summarizing inclusion and exclusion across four datasets: Group A—retrospective cases from Center A (Jan 2018–Jan 2025) for prompt optimization and framework testing; Group B—retrospective cases from Center B (May 2018–Mar 2025) for framework testing and validation; Group C—PubMed case reports (Jan 2024–Jan 2025) for framework testing and validation; and Group D—prospective cases from Center A (Feb–May 2025) for framework validation and human–AI interaction evaluation.


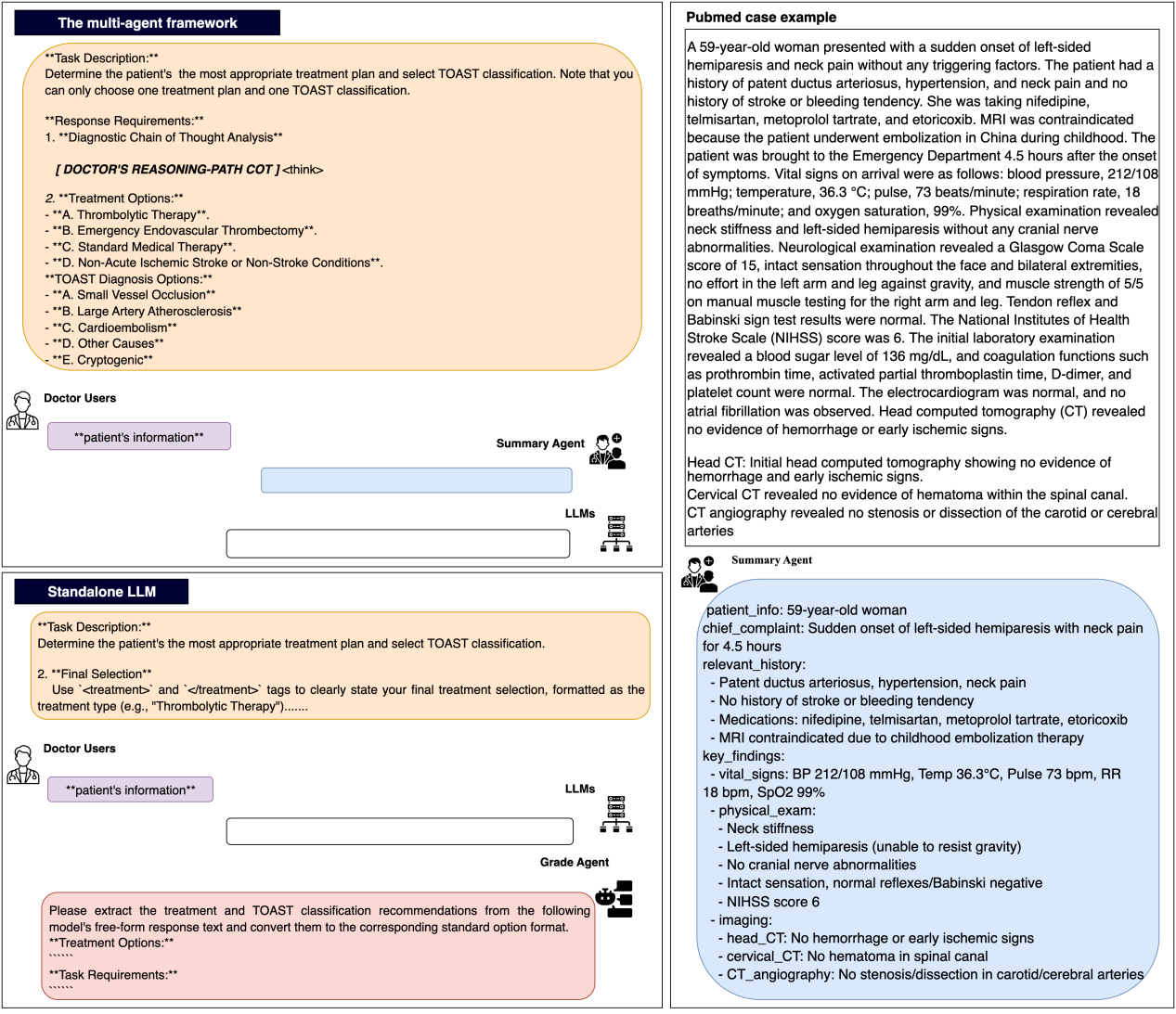


**Supplementary Figure S2. Visualization of interaction interfaces for standalone LLM and multi-agent framework augmented LLM.** Schematic illustrating the research workflow and output formats. The interface contrasts the multi-agent framework augmented LLM with standalone LLM. In first scenario, clinicians provide chain-of-thought reasoning using *<think>* tags and specify final treatment recommendation and TOAST classifications using *<treatment>* and *<diagnosis>* tags. A Summary Agent can optionally compress inputs to reduce token length. In second scenario, where responses are unconstrained, a Grade Agent extracts and standardizes answers. The right panel shows a PubMed case example demonstrating input information and structured output generation.


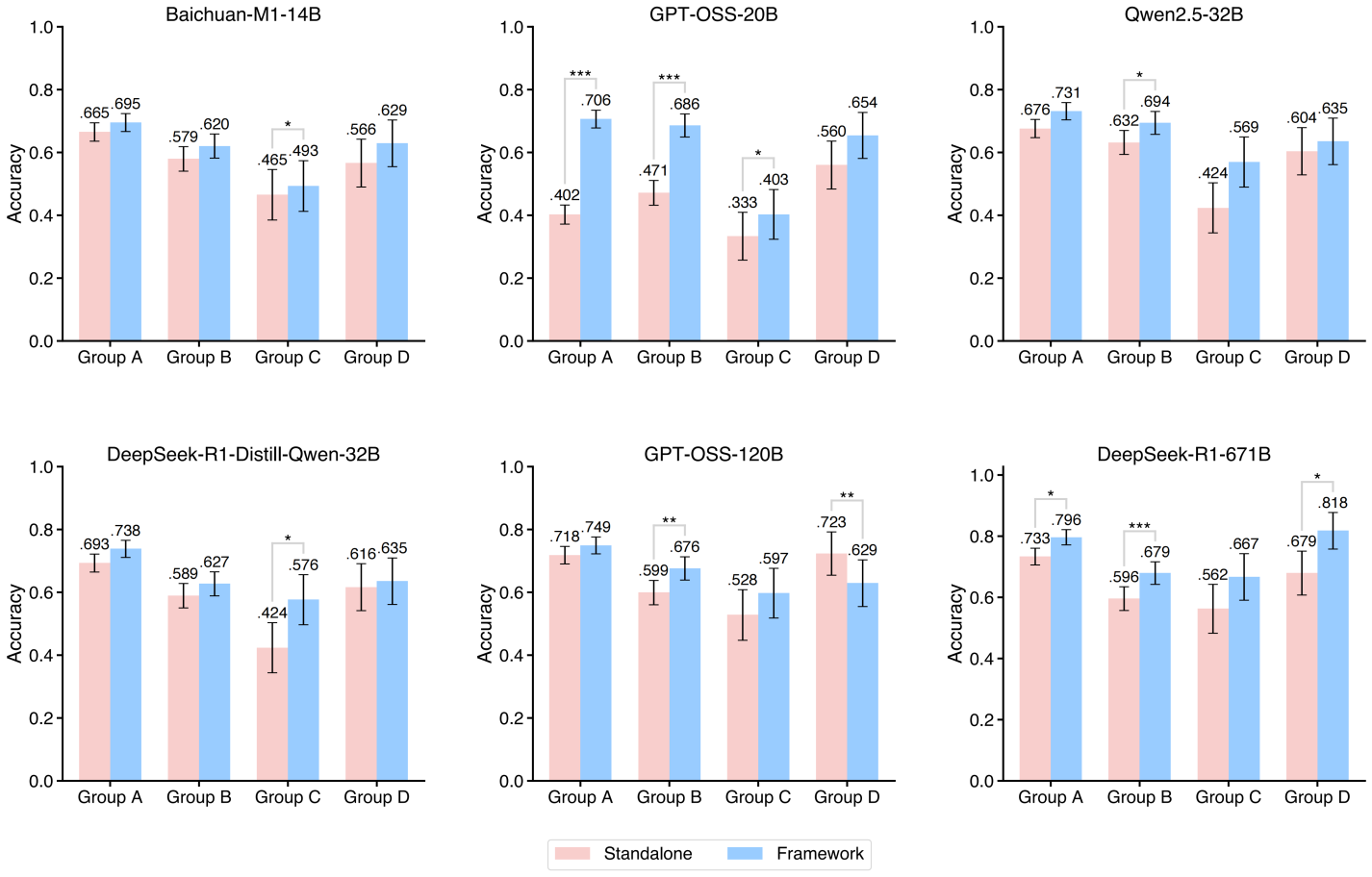


**Supplementary Material Figure S3. Multi-agent framework enhances accuracy of LLMs in TOAST classification.** ACC comparisons are shown for six LLMs of increasing scale (Baichuan-M1-14B, GPT-OSS-20B, Qwen2.5-32B, DeepSeek-R1-Distill-Qwen-32B, GPT-OSS-120B, and DeepSeek-R1-671B) across four evaluation groups (from group A to D). Within each group, paired bars represent standalone performance (pink) and Multi-agent framework augmented performance (blue).


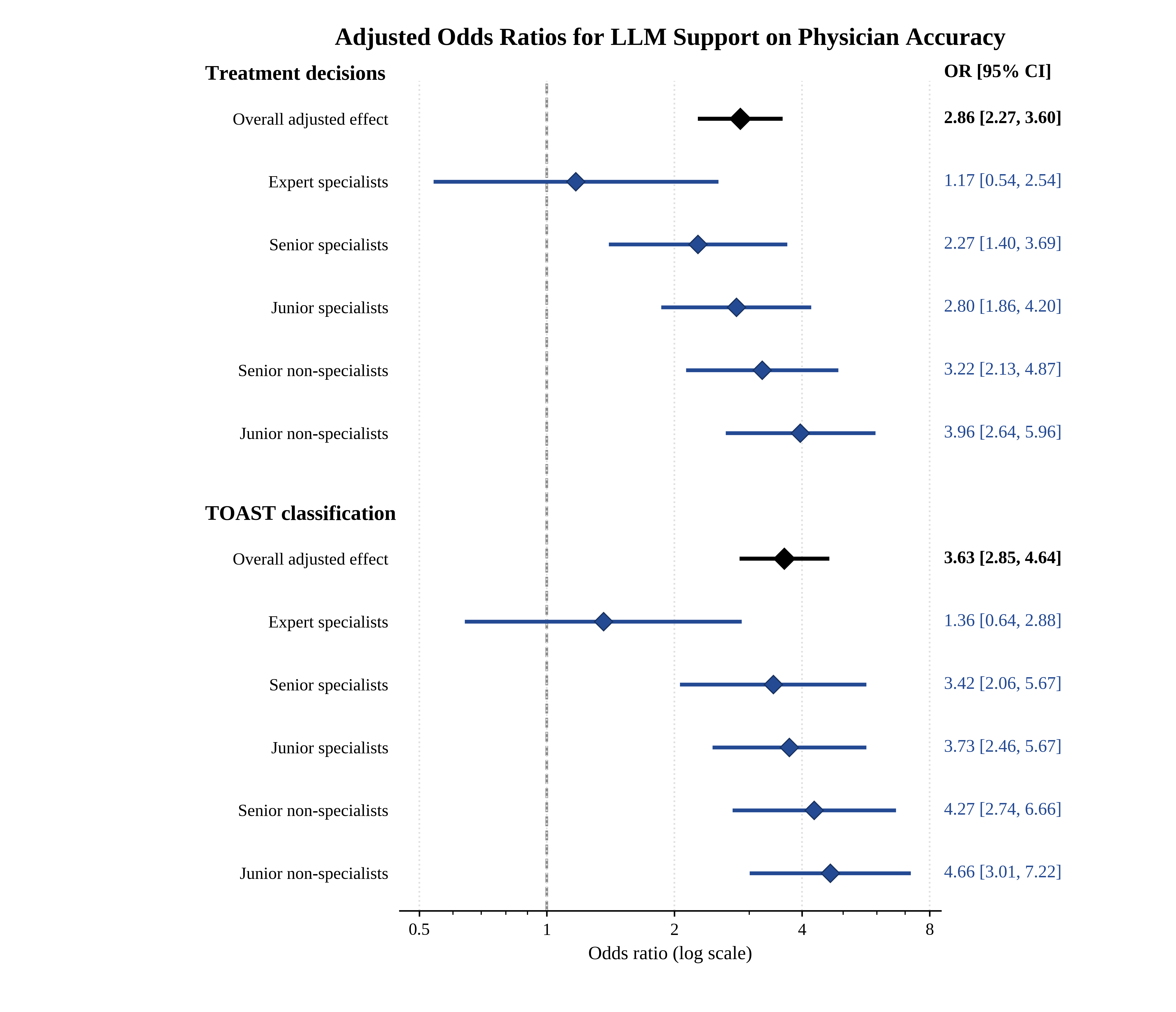


**Supplementary Material Figure S4. Forest plot of adjusted odds ratios for the association between LLM support and physician accuracy in the human–AI interaction experiment.** Separate binomial-logit generalized linear mixed models were fitted for treatment-decision correctness and TOAST-subtyping correctness. Models included LLM support, physician experience, and specialty as fixed effects, with crossed random intercepts for physician and case. Diamonds indicate adjusted odds ratios, horizontal lines indicate 95% CIs, and the dashed vertical line represents an odds ratio of 1. Values greater than 1 indicate higher odds of a correct decision with LLM support.
OR: odds ratio.

| **Models** | Model size | Developer | Type | Licence | Language support | Version date | Reference |
| --- | --- | --- | --- | --- | --- | --- | --- |
| Baichuan-M1^#^ | 14B | Baichuan-Inc | Medical | Apache License 2.0 | Chinese, English | 2025.2 | https://arxiv.org/abs/2502.12671 |
| GPT-OSS^&^ | 20B | OpenAI | General | Apache License 2.0 | Chinese, English | 2025.8 | https://cdn.openai.com/pdf/419b6906-9da6-406c-a19d-1bb078ac7637/oai_gpt-oss_model_card.pdf |
| Qwen2.5 | 32B | Alibaba | General | Apache License 2.0 | Chinese, English | 2024.9 | https://arxiv.org/abs/2412.15115 |
| DeepSeek-R1-Distill-Qwen | 32B | DeepSeek-AI | General | MIT License | Chinese, English | 2025.2 | https://arxiv.org/abs/2501.12948 |
| GPT-OSS^&^ | 120B | OpenAI | General | Apache License 2.0 | Chinese, English | 2025.8 | https://cdn.openai.com/pdf/419b6906-9da6-406c-a19d-1bb078ac7637/oai_gpt-oss_model_card.pdf |
| Deepseek-R1^†^ | 671B | DeepSeek‑AI | General | MIT License | Chinese, English | 2025.5 | https://arxiv.org/abs/2501.12948 |
| GPT-4o | Not disclosed | OpenAI | General | Proprietary (OpenAI Terms ) | Multilingual | 2024.5 | https://arxiv.org/abs/2410.21276 |

**Supplementary Table S1. The detailed information on the six LLMs used in this study.**

Detailed information on the six LLMs evaluated in this study, including model size, developer, application type, license, language support, version date, and reference source.

^#^Baichuan-M1 was released as a medical-domain–oriented model.

^&^GPT-OSS models (20B and 120B) were released by OpenAI with official model cards as technical references.

^†^ DeepSeek-R1 and DeepSeek-R1-Distill-Qwen were developed under the MIT licence, providing large-scale general-domain models with bilingual support.

**Supplementary Table S2. Outcome Metrics and Definitions**

| **Metric** | Definition | Unit of analysis | Used for | Reported in |
| --- | --- | --- | --- | --- |
| Accuracy | Proportion of predictions that match the clinical reference standard. For treatment recommendation, a prediction was correct if it matched the reference treatment category; for TOAST classification, a prediction was correct if it matched the reference TOAST subtype. | Case-model pair; physician-case decision | Primary performance metric for treatment recommendation and TOAST classification; physician decision correctness | Table 2; Figures 2, 4, and 6; Supplementary Figures S3-S4; Supplementary Tables S4-S7 |
| F1 score | Harmonic mean of precision and recall. For multiclass treatment recommendation and TOAST classification, F1 was calculated using one-vs-rest class-level evaluation and summarized as macro-average or task-level F1 where specified. | Class-level or macro-averaged case-model evaluation; physician-case decision where applicable | Multiclass performance evaluation, especially when class imbalance is present | Figure 3; Figure 6; Supplementary Tables S4-S7 |
| Sensitivity | Proportion of reference-positive cases correctly identified by the model in a one-vs-rest comparison. | Class-level one-vs-rest case-model pair; macro-average across classes | Detection of treatment or TOAST categories, including whether positive classes were missed | Supplementary Table S3 |
| Specificity | Proportion of reference-negative cases correctly identified as negative by the model in a one-vs-rest comparison. | Class-level one-vs-rest case-model pair; macro-average across classes | Assessment of false-positive tendency for treatment or TOAST categories | Supplementary Table S3 |
| PPV | Positive predictive value: proportion of model-positive predictions that were true positives. | Class-level one-vs-rest case-model pair; macro-average across classes | Reliability of positive treatment or TOAST predictions | Supplementary Table S3 |
| NPV | Negative predictive value: proportion of model-negative predictions that were true negatives. | Class-level one-vs-rest case-model pair; macro-average across classes | Reliability of negative treatment or TOAST predictions | Supplementary Table S3 |
| Risk difference | Absolute difference in accuracy or model-based marginal predicted probability between two conditions, such as with versus without LLM support, when reported. | Physician-case decision or condition-level comparison | Absolute effect-size interpretation in the physician study or condition comparisons | Results text / Figure 6, if retained |
| Odds ratio | Association between LLM support and correct physician decision estimated from a binomial-logit generalized linear mixed-effects model, with crossed random intercepts for physician and case. | Physician-case decision | Adjusted effect of framework augmentation on model-level correctness in Group A; adjusted effect of LLM support on physician treatment-decision and TOAST-subtyping correctness | Results text; Supplementary Figure S4 |
| Hallucination | Generation of unsupported, incorrect, or fabricated clinical, imaging, laboratory, or guideline-related information not present in the case materials or reference evidence. | Case-model output, counted as present or absent per output | Output safety and factual reliability assessment | Figure 5e; Supplementary Table S8 |
| Omission | Failure to include key decision-relevant information that was present in the case materials or reference evidence, such as time-window, contraindication, imaging, or clinical eligibility information. | Case-model output, counted as present or absent per output | Output completeness and safety assessment | Figure 5e; Supplementary Table S8 |
| Harmfulness / clinical safety rating | Clinician-rated potential clinical risk or safety quality of the output. Harmfulness was displayed as a 3-point rating, and clinical safety was summarized using factual accuracy, logical consistency, clinical relevance, and composite safety scores. | Case-model output or error event | Clinical safety assessment beyond accuracy | Figure 5c; Supplementary Table S8 |
| Instruction adherence | Whether the output followed the required response format, category constraints, and structured output requirements. | Case-model output | Output reliability and standardization of model responses | Figure 5d; Supplementary Table S8 |
| AIS-specific safety-critical error modes | Clinically interpretable error categories for reperfusion decision-making, including missed indication, overcalled indication, contraindication-related errors, time-window errors, imaging errors, severity/disabling-deficit errors, missed contraindication, and incomplete-evidence errors. | Case-level IVT/EVT error event; all evaluated cases and error cases as denominators | Clinical characterization of unsafe or incorrect treatment recommendations | Supplementary Table S9 (renumber as needed after final supplementary-table ordering) |

| Models | Tasks | Standalone LLM | | | | Multi-agent Framework | | | |
| --- | --- | --- | --- | --- | --- | --- | --- | --- | --- |
|  |  | Sensitivity | Specificity | PPV | NPV | Sensitivity | Specificity | PPV | NPV |
| Baichuan-M1-14B | Treatment | 0.434 (0.411–0.456) | 0.857 (0.850–0.863) | 0.533 (0.502–0.558) | 0.858 (0.850–0.864) | 0.529 (0.506–0.554) | 0.888 (0.883–0.896) | 0.682 (0.652–0.716) | 0.896 (0.890–0.903) |
|  | TOAST | 0.314 (0.293–0.337) | 0.880 (0.873–0.886) | 0.301 (0.273–0.330) | 0.875 (0.869–0.881) | 0.360 (0.332–0.388) | 0.898 (0.892–0.905) | 0.304 (0.280–0.330) | 0.886 (0.881–0.892) |
| GPT-OSS-20B | Treatment | 0.356 (0.334–0.379) | 0.858 (0.852–0.864) | 0.413 (0.389–0.435) | 0.826 (0.821–0.831) | 0.542 (0.517–0.568) | 0.886 (0.880–0.891) | 0.567 (0.540–0.594) | 0.868 (0.862–0.873) |
|  | TOAST | 0.205 (0.187–0.222) | 0.888 (0.881–0.894) | 0.221 (0.197–0.249) | 0.852 (0.848–0.856) | 0.323 (0.299–0.348) | 0.900 (0.893–0.905) | 0.305 (0.279–0.335) | 0.893 (0.888–0.898) |
| Qwen2.5-32B | Treatment | 0.472 (0.450–0.497) | 0.876 (0.870–0.883) | 0.504 (0.481–0.527) | 0.867 (0.861–0.873) | 0.530 (0.509–0.550) | 0.892 (0.886–0.898) | 0.724 (0.693–0.755) | 0.897 (0.890–0.903) |
|  | TOAST | 0.271 (0.251–0.292) | 0.870 (0.864–0.876) | 0.331 (0.288–0.377) | 0.872 (0.865–0.878) | 0.357 (0.330–0.384) | 0.898 (0.892–0.904) | 0.333 (0.303–0.362) | 0.895 (0.889–0.902) |
| DeepSeek-Distill-Qwen-32B | Treatment | 0.464 (0.442–0.488) | 0.873 (0.867–0.881) | 0.524 (0.502–0.548) | 0.868 (0.862–0.876) | 0.557 (0.535–0.579) | 0.899 (0.893–0.905) | 0.778 (0.750–0.804) | 0.915 (0.909–0.921) |
|  | TOAST | 0.334 (0.311–0.355) | 0.890 (0.883–0.895) | 0.287 (0.264–0.314) | 0.883 (0.877–0.888) | 0.389 (0.365–0.414) | 0.907 (0.901–0.913) | 0.313 (0.296–0.333) | 0.894 (0.890–0.900) |
| GPT-OSS-120B | Treatment | 0.620 (0.600–0.644) | 0.916 (0.910–0.922) | 0.638 (0.616–0.662) | 0.904 (0.898–0.911) | 0.711 (0.686–0.732) | 0.942 (0.937–0.947) | 0.796 (0.770–0.821) | 0.937 (0.931–0.943) |
|  | TOAST | 0.387 (0.362–0.414) | 0.903 (0.897–0.909) | 0.314 (0.282–0.353) | 0.891 (0.886–0.896) | 0.473 (0.443–0.505) | 0.919 (0.913–0.924) | 0.382 (0.359–0.406) | 0.902 (0.896–0.907) |
| DeepSeek-R1-671B | Treatment | 0.523 (0.501–0.547) | 0.887 (0.881–0.893) | 0.649 (0.623–0.674) | 0.900 (0.893–0.906) | 0.682 (0.658–0.704) | 0.934 (0.929–0.939) | 0.873 (0.852–0.891) | 0.949 (0.944–0.953) |
|  | TOAST | 0.420 (0.395–0.447) | 0.903 (0.898–0.909) | 0.327 (0.297–0.372) | 0.894 (0.888–0.898) | 0.535 (0.503–0.569) | 0.934 (0.929–0.939) | 0.425 (0.400–0.451) | 0.914 (0.909–0.920) |

**Supplementary Table S3. Performance of LLMs in treatment recommendation and TOAST classification in all groups.**

Note. Values are macro-averaged one-vs-rest metrics, with 95% CIs shown in parentheses. LLM: large language model; NPV: negative predictive value; PPV: positive predictive value; TOAST: Trial of Org 10172 in Acute Stroke Treatment

**Supplementary Table S4. Performance of LLMs in treatment recommendation and TOAST classification in Group A.**

| **Models** | ACC | Standalone LLM | Multi-agent Framework | P value | F1 Score | Standalone LLM | Multi-agent Framework | P value |
| --- | --- | --- | --- | --- | --- | --- | --- | --- |
| Baichuan-M1-14B | ACC of Treatment | 0.546 (0.517, 0.576) | 0.687 (0.658, 0.713) | <0.001 | F1 of Treatment | 0.707 (0.682, 0.731) | 0.814 (0.794, 0.832) | 0.760 |
|  | ACC of TOAST | 0.665 (0.637, 0.695) | 0.695 (0.669, 0.724) | 0.001 | F1 of TOAST | 0.303 (0.277, 0.328) | 0.275 (0.247, 0.351) | 0.796 |
| GPT-OSS-20B | ACC of Treatment | 0.433 (0.400, 0.465) | 0.487 (0.456, 0.518) | <0.001 | F1 of Treatment | 0.604 (0.571, 0.635) | 0.655 (0.627, 0.682) | <0.001 |
|  | ACC of TOAST | 0.508 (0.472, 0.544) | 0.706 (0.681, 0.735) | <0.001 | F1 of TOAST | 0.224 (0.200, 0.248) | 0.235 (0.209, 0.299) | <0.001 |
| Qwen2.5-32B | ACC of Treatment | 0.574 (0.543, 0.603) | 0.697 (0.673, 0.725) | 0.009 | F1 of Treatment | 0.729 (0.704, 0.753) | 0.822 (0.804, 0.84) | <0.001 |
|  | ACC of TOAST | 0.678 (0.650, 0.708) | 0.731 (0.705, 0.76) | <0.001 | F1 of TOAST | 0.275 (0.241, 0.314) | 0.307 (0.271, 0.406) | 0.090 |
| DeepSeek-Distill-Qwen-32B | ACC of Treatment | 0.609 (0.579, 0.637) | 0.762 (0.735, 0.788) | <0.001 | F1 of Treatment | 0.757 (0.733, 0.778) | 0.865 (0.847, 0.882) | <0.001 |
|  | ACC of TOAST | 0.694 (0.665, 0.723) | 0.738 (0.709, 0.764) | <0.001 | F1 of TOAST | 0.347 (0.315, 0.383) | 0.354 (0.319, 0.388) | 0.004 |
| GPT-OSS-120B | ACC of Treatment | 0.737 (0.710, 0.763) | 0.851 (0.829, 0.872) | 0.005 | F1 of Treatment | 0.849 (0.830, 0.865) | 0.92 (0.907, 0.932) | <0.001 |
|  | ACC of TOAST | 0.731 (0.704, 0.76) | 0.749 (0.722, 0.777) | 0.823 | F1 of TOAST | 0.426 (0.367, 0.481) | 0.417 (0.373, 0.462) | 0.550 |
| DeepSeek-R1-671B | ACC of Treatment | 0.687 (0.658, 0.714) | 0.847 (0.826, 0.868) | <0.001 | F1 of Treatment | 0.814 (0.794, 0.833) | 0.917 (0.905, 0.929) | <0.001 |
|  | ACC of TOAST | 0.733 (0.705, 0.763) | 0.796 (0.770, 0.820) | 0.118 | F1 of TOAST | 0.420 (0.375, 0.459) | 0.474 (0.427, 0.520) | 0.336 |

The table presents ACC (accuracy) and F1 scores for standalone and framework-augmented LLMs in Group A, with corresponding P values for paired comparisons. Values are reported as point estimates, with 95% confidence intervals (CIs) shown in parentheses. ACC, accuracy.

**Supplementary Table S5. Performance of LLMs in treatment recommendation and TOAST classification in Group B.**

| **Models** | ACC | Standalone LLM | Multi-agent Framework | P value | F1 score | Standalone LLM | Multi-agent Framework | P value |
| --- | --- | --- | --- | --- | --- | --- | --- | --- |
| Baichuan-M1-14B | ACC of Treatment | 0.595 (0.559, 0.631) | 0.684 (0.652, 0.718) | <0.001 | F1 of Treatment | 0.746(0.717, 0.774) | 0.812 (0.789, 0.836) | 0.190 |
|  | ACC of TOAST | 0.583 (0.545, 0.623) | 0.620 (0.581, 0.656) | 0.027 | F1 of TOAST | 0.267 (0.229, 0.308) | 0.262 (0.223, 0.302) | 0.044 |
| GPT-OSS-20B | ACC of Treatment | 0.459 (0.423, 0.499) | 0.592 (0.555, 0.632) | <0.001 | F1 of Treatment | 0.629 (0.594, 0.665) | 0.744 (0.714, 0.775) | <0.001 |
|  | ACC of TOAST | 0.576 (0.534, 0.616) | 0.686 (0.651, 0.723) | 0.001 | F1 of TOAST | 0.232 (0.200, 0.263) | 0.27 (0.228, 0.316) | <0.001 |
| Qwen2.5-32B | ACC of Treatment | 0.587 (0.551, 0.623) | 0.671 (0.638, 0.706) | 0.022 | F1 of Treatment | 0.740 (0.710, 0.768) | 0.803 (0.779, 0.828) | 0.110 |
|  | ACC of TOAST | 0.633 (0.592, 0.674) | 0.694 (0.656, 0.728) | 0.683 | F1 of TOAST | 0.280 (0.228, 0.335) | 0.296 (0.247, 0.344) | 0.204 |
| DeepSeek-Distill-Qwen-32B | ACC of Treatment | 0.587 (0.551, 0.623) | 0.678 (0.644, 0.714) | 0.382 | F1 of Treatment | 0.740 (0.710, 0.768) | 0.808 (0.783, 0.833) | <0.001 |
|  | ACC of TOAST | 0.593 (0.554, 0.629) | 0.627 (0.591, 0.664) | 0.505 | F1 of TOAST | 0.309 (0.252, 0.365) | 0.26 (0.225, 0.294) | 0.004 |
| GPT-OSS-120B | ACC of Treatment | 0.699 (0.667, 0.732) | 0.798 (0.770, 0.828) | <0.001 | F1 of Treatment | 0.823 (0.800, 0.845) | 0.887 (0.870, 0.906) | 0.042 |
|  | ACC of TOAST | 0.622 (0.583, 0.658) | 0.676 (0.640, 0.712) | 0.752 | F1 of TOAST | 0.315 (0.269, 0.359) | 0.353 (0.300, 0.400) | 0.004 |
| DeepSeek-R1-671B | ACC of Treatment | 0.671 (0.638, 0.705) | 0.813 (0.785, 0.841) | <0.001 | F1 of Treatment | 0.803 (0.779, 0.827) | 0.897 (0.880, 0.913) | 0.176 |
|  | ACC of TOAST | 0.600 (0.562, 0.634) | 0.679 (0.646, 0.715) | 0.131 | F1 score of TOAST | 0.351 (0.288, 0.404) | 0.39 (0.334, 0.442) | <0.001 |

The table reports ACC and F1 scores for standalone and framework-augmented LLMs in Group B, with corresponding P values for paired comparisons. Values are presented as point estimates, with 95% CIs shown in parentheses.

**Supplementary Table S6. Performance of LLMs in treatment recommendation and TOAST classification in Group C.**

| **Models** | ACC | Standalone LLM | Multi-agent Framework | P value | F1 score | Standalone LLM | Multi-agent Framework | P value |
| --- | --- | --- | --- | --- | --- | --- | --- | --- |
| Baichuan-M1-14B | ACC of Treatment | 0.507 (0.424, 0.59) | 0.667 (0.583, 0.743) | 0.011 | F1 score of Treatment | 0.673 (0.595, 0.742) | 0.800 (0.737, 0.853) | 0.872 |
|  | ACC of TOAST | 0.549 (0.459, 0.631) | 0.568 (0.480, 0.656) | 0.480 | F1 score of TOAST | 0.444 (0.362, 0.517) | 0.474 (0.374, 0.581) | 0.942 |
| GPT-OSS-20B | ACC of Treatment | 0.497 (0.42, 0.573) | 0.671 (0.594, 0.748) | 0.111 | F1 score of Treatment | 0.664 (0.591, 0.729) | 0.803 (0.745, 0.856) | 0.044 |
|  | ACC of TOAST | 0.400 (0.317, 0.483) | 0.468 (0.387, 0.565) | 1.0 | F1 score of TOAST | 0.300 (0.232, 0.361) | 0.419 (0.332, 0.507) | 0.378 |
| Qwen2.5-32B | ACC of Treatment | 0.618 (0.542, 0.701) | 0.674 (0.590, 0.743) | 0.689 | F1 score of Treatment | 0.764 (0.703, 0.824) | 0.805 (0.742, 0.853) | 0.018 |
|  | ACC of TOAST | 0.488 (0.400, 0.576) | 0.656 (0.576, 0.736) | 0.480 | F1 score of TOAST | 0.369 (0.296, 0.441) | 0.560 (0.447, 0.667) | 0.126 |
| DeepSeek-Distill-Qwen-32B | ACC of Treatment | 0.528 (0.451, 0.611) | 0.688 (0.604, 0.764) | 0.153 | F1 score of Treatment | 0.691 (0.622, 0.759) | 0.815 (0.753, 0.866) | 0.042 |
|  | ACC of TOAST | 0.496 (0.415, 0.585) | 0.664 (0.584, 0.744) | 1.0 | F1 score of TOAST | 0.393 (0.316, 0.467) | 0.534 (0.452, 0.608) | 0.498 |
| GPT-OSS-120B | ACC of Treatment | 0.597 (0.514, 0.674) | 0.750 (0.681, 0.826) | 0.677 | F1 score of Treatment | 0.748 (0.679, 0.805) | 0.857 (0.810, 0.905) | 0.002 |
|  | ACC of TOAST | 0.608 (0.528, 0.688) | 0.688 (0.616, 0.768) | 1.0 | F1 score of TOAST | 0.513 (0.413, 0.613) | 0.590 (0.483, 0.696) | 0.852 |
| DeepSeek-R1-671B | ACC of Treatment | 0.646 (0.562, 0.722) | 0.729 (0.653, 0.799) | 0.844 | F1 score of Treatment | 0.785 (0.720, 0.839) | 0.843 (0.790, 0.888) | 0.022 |
|  | ACC of TOAST | 0.648 (0.568, 0.728) | 0.768 (0.696, 0.840) | 1.0 | F1 score of TOAST | 0.558 (0.443, 0.663) | 0.708 (0.556, 0.814) | 0.460 |

The table summarizes ACC and F1 scores for standalone and framework-augmented LLMs in Group C, with corresponding P values for paired comparisons. Values are reported as point estimates, with 95% CIs shown in parentheses. **In Group C, GPT-4o gained accuracy in treatment recommendation (+14.9%, 0.750 vs. 0.653) but showed a decline in TOAST classification (−6.7%, 0.486 vs. 0.521).**

**Supplementary Table S7. Performance of LLMs in treatment recommendation and TOAST classification in Group D.**

| **Models** | ACC | Standalone LLM | Multi-agent Framework | P value | F1 score | Standalone LLM | Multi-agent Framework | P value |
| --- | --- | --- | --- | --- | --- | --- | --- | --- |
| Baichuan-M1-14B | ACC of Treatment | 0.665 (0.584, 0.739) | 0.820 (0.758, 0.876) | 0.780 | F1 of Treatment | 0.799 (0.737, 0.85) | 0.901 (0.862, 0.934) | 0.228 |
|  | ACC of TOAST | 0.566 (0.491, 0.648) | 0.629 (0.547, 0.704) | 0.022 | F1 of TOAST | 0.255 (0.211, 0.461) | 0.271 (0.224, 0.41) | 0.010 |
| GPT-OSS-20B | ACC of Treatment | 0.633 (0.557, 0.709) | 0.547 (0.466, 0.627) | 0.034 | F1 of Treatment | 0.775 (0.715, 0.83) | 0.707 (0.636, 0.771) | <0.001 |
|  | ACC of TOAST | 0.597 (0.517, 0.671) | 0.654 (0.579, 0.730) | 0.080 | F1 of TOAST | 0.312 (0.261, 0.455) | 0.249 (0.207, 0.329) | 0.474 |
| Qwen2.5-32B | ACC of Treatment | 0.727 (0.652, 0.789) | 0.783 (0.714, 0.851) | 0.025 | F1 of Treatment | 0.842 (0.789, 0.882) | 0.878 (0.833, 0.919) | 0.014 |
|  | ACC of TOAST | 0.604 (0.528, 0.679) | 0.635 (0.56, 0.711) | <0.001 | F1 of TOAST | 0.257 (0.216, 0.467) | 0.306 (0.251, 0.548) | 0.004 |
| DeepSeek-Distill-Qwen-32B | ACC of Treatment | 0.652 (0.578, 0.727) | 0.845 (0.789, 0.901) | <0.001 | F1 of Treatment | 0.789 (0.732, 0.842) | 0.916 (0.882, 0.948) | 0.258 |
|  | ACC of TOAST | 0.624 (0.548, 0.694) | 0.635 (0.560, 0.711) | 0.052 | F1 of TOAST | 0.298 (0.25, 0.542) | 0.371 (0.258, 0.548) | 0.208 |
| GPT-OSS-120B | ACC of Treatment | 0.870 (0.814, 0.919) | 0.839 (0.776, 0.894) | 0.579 | F1 of Treatment | 0.93 (0.897, 0.958) | 0.912 (0.874, 0.944) | 0.968 |
|  | ACC of TOAST | 0.747 (0.682, 0.818) | 0.629 (0.553, 0.704) | 0.027 | F1 of TOAST | 0.422 (0.377, 0.698) | 0.328 (0.263, 0.555) | 0.638 |
| DeepSeek-R1-671B | ACC of Treatment | 0.770 (0.702, 0.832) | 0.882 (0.826, 0.932) | <0.001 | F1 of Treatment | 0.87 (0.825, 0.908) | 0.937 (0.905, 0.965) | <0.001 |
|  | ACC of TOAST | 0.679 (0.604, 0.748) | 0.818 (0.755, 0.881) | 0.683 | F1 of TOAST | 0.395 (0.35, 0.644) | 0.479 (0.431, 0.628) | 0.016 |

The table shows ACC (Accuracy) and F1 scores for standalone and framework-augmented LLMs in Group D, with corresponding P values for paired comparisons. Values are presented as point estimates, with 95% CIs shown in parentheses.

**Supplementary Table S8. Clinical safety indicators of LLMs during preliminary deployment.**

| **Models** | Setting | Instruction coherence | 1: Factual Accuracy | 2: Logical Consistency | 3: Clinical Relevance | Composite Evaluation Score across items 1-3 | Hallucination | Omission |
| --- | --- | --- | --- | --- | --- | --- | --- | --- |
| Baichuan-M1-14B | Multi-agent Framework | 0.994 | 3.995 | 4.390 | 3.696 | 4.027 | 18.9% | 24.3% |
|  | Standalone LLM | 0.889 | 3.576 | 4.143 | 3.320 | 3.680 | 38.3% | 42.5% |
| GPT-OSS-20B | Multi-agent Framework | 0.801 | 3.491 | 3.723 | 2.853 | 3.356 | 33.6% | 40.4% |
|  | Standalone LLM | 0.573 | 2.895 | 3.296 | 2.578 | 2.923 | 53.2% | 61.6% |
| Qwen2.5-32B | Multi-agent Framework | 0.997 | 4.002 | 4.383 | 3.627 | 4.004 | 26.1% | 29.8% |
|  | Standalone LLM | 0.916 | 3.697 | 4.189 | 3.321 | 3.736 | 34.6% | 39.0% |
| DeepSeek-R1-Distill-Qwen32B | Multi-agent Framework | 0.994 | 3.988 | 4.422 | 3.616 | 4.007 | 22.3% | 23.7% |
|  | Standalone LLM | 0.928 | 3.596 | 4.193 | 3.317 | 3.702 | 35.2% | 37.5% |
| GPT-OSS-120B | Multi-agent Framework | 0.998 | 4.227 | 4.666 | 4.0 | 4.298 | 11.7% | 14.0% |
|  | Standalone LLM | 0.978 | 4.044 | 4.622 | 3.833 | 4.166 | 20.0% | 23.4% |
| DeepSeek-R1-671B | Multi-agent Framework | 0.998 | 4.280 | 4.760 | 4.027 | 4.355 | 10.9% | 14.7% |
|  | Standalone LLM | 0.966 | 3.903 | 4.484 | 3.677 | 4.021 | 20.4% | 26.9% |

The composite evaluation score was calculated as the mean of factual accuracy, logical consistency, and clinical relevance ratings. Hallucination and omission rates were calculated as the proportion of outputs containing at least one hallucination or omission event.

**Supplementary Table S9. Error-mode analysis of incorrect recommendations generated by the DeepSeek-R1 model during preliminary deployment.**

| **Error category** | Definition | Standalone | | Multi-agent Framework | |
| --- | --- | --- | --- | --- | --- |
|  |  | All evaluated cases, % | Error cases, % | All evaluated cases, % | Error cases, % |
| Missed indication | Reference standard supports IVT/EVT, but the model failed to recommend reperfusion. | 9.9% | 28.5% | 5.5% | 17.7% |
| Overcalled despite contraindication | Model noted or had access to contraindication/risk but still recommended aggressive reperfusion. | 9.6% | 27.8% | 7.6% | 24.6% |
| Time window error | Incorrect use of onset/last known well/arrival/imaging timing caused under- or over-recommendation. | 7.5% | 21.7% | 8.9% | 28.6% |
| Imaging error | Misread or misweighted CTA/CTP/NCCT/LVO evidence, mainly affecting EVT. | 4.7% | 13.5% | 3.4% | 11.1% |
| Overcalled indication | Insufficient indications or evidence, yet the model recommended reperfusion. | 3.7% | 10.8% | 6.0% | 19.4% |
| Severity/disabling deficit error | Misjudged NIHSS severity or disabling deficit status. | 0.9% | 2.7% | 0.4% | 1.4% |
| Missed contraindication | Contraindication or high-risk factor was present but not recognized or incorporated. | 0.3% | 0.9% | 0.1% | 0.5% |
| Evidence absent/incomplete | Acted on absent or incomplete information as if sufficient. | 0.1% | 0.3% | 0.1% | 0.5% |
